# Where Do Children Look When Watching Videos With Same-Language Subtitles?

**DOI:** 10.1177/09567976251325789

**Published:** 2025-04-02

**Authors:** Anastasiya Lopukhina, Walter J. B. van Heuven, Rebecca Crowley, Kathleen Rastle

**Affiliations:** 1 Department of Psychology, Royal Holloway, University of London; 2 School of Psychology, University of Nottingham

**Keywords:** reading, same-language subtitles, closed captions, primary-school children, eye tracking

## Abstract

Influential campaigns in the United Kingdom and the United States have argued that same-language television subtitles may help children learn to read. In this study, we investigated the extent to which primary-school children pay attention to and read subtitles and whether this is related to their reading proficiency. We tracked the eye movements of 180 British children in Years 1 to 6 who watched videos with and without subtitles. Results showed that attention to subtitles was associated with reading proficiency: Superior readers were more likely to look at subtitles than less proficient readers and spent more time on them. When children looked at words in the subtitles, they showed evidence of reading them. We conclude that some degree of reading fluency may be necessary before children pay attention to subtitles. However, by the third or fourth year of reading instruction, most children read sufficiently quickly to follow same-language subtitles and potentially learn from them.

## Introduction

Learning to read is the most important outcome of primary-school education. Reading is an important foundation for individual health, wealth, and prosperity and is a vital driver of economic development. Yet low literacy remains a staggering global challenge: Approximately 57% of children in low- and middle-income countries are unable to read with basic comprehension by age 10 (World Bank, 2019); the same is true for 6% of children aged 10 in the European Union (Mullis et al., 2023).

Becoming a skilled reader requires high-quality instruction but also many years of reading practice (Castles et al., 2018). Thus, barriers to literacy include a lack of engagement in independent reading and limited access to books in some populations (Cole et al., 2022). For example, 49% of England’s 10-year-old pupils report reading less than 30 min per day outside of school, with poorer readers tending to have fewer books at home and showing less enjoyment of reading (Lindorff et al., 2024). One appealing solution has been proposed by public campaigns such as Turn on the Subtitles (https://turnonthesubtitles.org/) and CaptionsON (https://www.captionson.org/). They argue that turning on same-language subtitles while children are watching television can dramatically improve children’s reading outcomes, as this turns “television time” into “reading time.” These campaigns have been highly successful in drawing media stakeholders, celebrities, and even the British Prime Minister (Hansard, 2020) into the effort to turn on default subtitling for children’s television programs. However, whereas substantial research has investigated how skilled adult readers engage with subtitles (e.g., d’Ydewalle et al., 1991; Kruger & Steyn, 2014; Perego et al., 2010), and how subtitles improve understanding in hard-of-hearing populations (Yoon & Kim, 2011) and second-language learners (Montero Perez et al., 2013; Vanderplank, 2016; see Gernsbacher, 2015, for a review), research is sparse on how same-language subtitles influence children’s reading.

One basic question is whether children even pay attention to same-language subtitles, or whether this requires some degree of prior reading proficiency. Though research monitoring eye movements indicates that adults read same-language subtitles (e.g., d’Ydewalle et al., 1991), we are unaware of any research that has investigated whether this is the case for children learning to read. The closest research to this scenario was a study in which adults in rural India who had completed their schooling but who were classified as weak readers watched Bollywood clips with and without same-language subtitles (Arjun et al., 2022). Their gaze was tracked with a rudimentary eye tracker sampling at 60 Hz. Arjun et al. (2022) reported that participants spent more time looking at the subtitle region and that a greater proportion of fixations were in that region when the video included same-language subtitles than when it did not. These data suggest that even poor readers may try to read same-language subtitles. However, in addition to the severe limitations in data acquisition posed by the sampling rate, the statistical reporting in this study was underspecified, making it difficult to evaluate the findings.

Indirect evidence that children pay attention to and read same-language subtitles comes from intervention studies probing whether experience with subtitles improves children’s reading skills. These studies show a positive influence of subtitles on children’s reading, but they are characterized by significant methodological limitations. One set of studies showed that a small number of sessions watching subtitled videos improved word recognition scores of English-speaking children between the ages of 7 and 10 (Linebarger, 2001; Linebarger et al., 2010); however, these studies are likely to be underpowered given the complex, between-groups interaction designs implemented (Brysbaert, 2019). In another study, daily exposure to subtitled videos over a 6- to 8-week period led to apparent gains in the reading comprehension and vocabulary of English-speaking children between the ages of 8 and 11 (Parkhill & Davey, 2014); however, this study did not include a control group to measure the gains against. Finally, researchers reported significant gains in decoding skill in Hindi-speaking children aged 6 to 14 in India who reported regular viewing of a subtitled program featuring Bollywood songs compared to children who reported infrequent or no viewing of the program (Kothari & Bandyopadhyay, 2014). However, the assignment of children into groups of those who watched the program versus those who did not was based on retrospective self-report. This design feature means that we cannot have confidence that the two groups were equivalent; for example, perhaps children who chose to watch the target program did so because their families were wealthy enough to afford a television. This study also lacked a measure of the baseline reading skills of the postintervention groups.

The present study sought to determine whether primary-school children pay attention to same-language subtitles, whether they show evidence of linguistic processing of these subtitles, and whether these outcomes are associated with children’s reading proficiency. We ran a well-powered, preregistered experiment to compare eye movements when English-speaking primary-school children in Years 1 to 6 (ages 5–11) and adults (ages 19–42) were watching short videos with and without subtitles. We hypothesized that some degree of reading proficiency would be required before children engaged with the subtitles and showed evidence of linguistic processing. We also sought to find out whether subtitles affect children’s understanding of video content, because it is important to demonstrate that a default subtitling initiative would not be distracting for the youngest children and impair comprehension of story details, as observed by Linebarger (2001). The adults were included as a manipulation check (in case no effects of subtitles were observed for the children) and also to help us understand how viewing behavior changes beyond Year 6; hence, data involving adult participants are reported only in the Supplemental Material available online.

### Research Transparency Statement

#### General disclosures

**Conflicts of interest:** The authors declare no conflicts of interest. **Funding:** This work was supported by a research grant from the Nuffield Foundation (FR-23450). **Artificial intelligence:** No artificial-intelligence-assisted technologies were used in this research or the creation of this article. **Ethics:** This research complies with the Declaration of Helsinki (2023) and received approval from a local ethics board (Reference No. 3484).

#### Study disclosures

**Preregistration:** The hypotheses, methods, and analysis plan were preregistered (https://doi.org/10.17605/OSF.IO/NVXFM) on March 27, 2023, prior to data collection, which began on March 28, 2023. There were minor deviations from the preregistration (for details, see Table S1 in the Supplemental Material). **Materials:** Some materials are publicly available (https://doi.org/10.17605/OSF.IO/PQYWF). The videos cannot be shared publicly because they are copyright protected; however, they can be made available upon request by contacting Kathleen Rastle (kathy.rastle@rhul.ac.uk). We also report the titles of the movies and the time stamps of the episodes and share the subtitles so that the exact episodes can be identified. **Data:** All raw data are publicly available (https://doi.org/10.17605/OSF.IO/5TD87). **Analysis scripts:** All analysis scripts are publicly available (https://doi.org/10.17605/OSF.IO/5BDAK). **Computational reproducibility:** The computational reproducibility of the results has been independently confirmed by the journal’s STAR team.

## Method

### Data availability

The experiment was preregistered (https://doi.org/10.17605/OSF.IO/NVXFM). Stimulus materials, data, analysis scripts, and Supplemental Material are available on the Open Science Framework (https://osf.io/uk8bt/).

### Design

The experiment used a within-participant design with two conditions: videos with subtitles versus videos without subtitles. The assignment of video to condition was counterbalanced via a Latin-square design resulting in six experimental lists. Each participant encountered all videos in a randomized order.

### Participants

Participants were 190 children (99 girls; age range 5–11 years) and 33 adults from Royal Holloway, University of London (24 females; age range 19–42 years). Virtually all child participants came from three state primary schools belonging to a multi-school consortium in the south of England; two other participants from different schools were tested in the laboratory. All participants were native-English speakers with normal or corrected-to-normal vision and hearing. The children had no special educational needs, and the adults reported no history of language or literacy impairment. All children were tested in England’s summer term, running from April through July. Ten children and three adults had to be excluded for the following reasons: calibration problems resulting in poor data quality (11 participants); excessive blinking (1 participant); unwillingness to proceed (1 participant).

In total, 180 children (30 per school-year group) and 30 adults were included in the analysis (see Table 1 for demographic information). We took the approach to power recommended by Brysbaert and Stevens (2018), who suggested having a minimum of 1,600 observations per condition (i.e., the number of items multiplied by the number of participants) in response-time studies using repeated measures to achieve 80% power for detecting typical effect sizes in psychology of *d* = 0.4. With 30 participants per group, we had 4,500 to 6,450 observations per condition (i.e., the number of subtitles in a pair of videos in one condition multiplied by the number of participants in each year group), substantially exceeding the minimum benchmark suggested by Brysbaert and Stevens (2018). Of the children in the sample, 17% were classed as disadvantaged according to the “pupil premium” metric used in England (House of Commons Library, 2023). Further, 174 children in the sample had passed the phonics screening test, a nonspeeded test of decoding knowledge given to all pupils in England toward the end of Year 1, in which children are asked to read 20 simple words and 20 simple nonwords aloud. Two participants in Year 4 had received scores below the pass mark of 32 out of 40, and no data were available for four participants.

**Table 1.** Demographic Information

School year	Age (mean and range)	Gender (females/males)	Number of disadvantaged pupils	Phonics screening test (mean and range)	TOWRE raw scores (mean and range)	BPVS raw scores (mean and range)
Year 1	5.7 (5–6)	16/14	6	38 (33–40)	55.3 (10–106)	92.4 (65–135)
Year 2	6.9 (6–8)	16/14	6	38 (34–40)	86.7 (39–122)	106.3 (93–131)
Year 3	7.7 (7–8)	17/13	3	39 (32–40)	95.7 (59–123)	118.7 (99–135)
Year 4	8.6 (8–9)	16/14	5	37 (22–40)	100.1 (45–132)	125.9 (98–153)
Year 5	9.8 (9–10)	15/15	4	37 (33–40)	113.5 (63–149)	135.4 (109–154)
Year 6	10.7 (10–11)	16/14	6	37 (32–40)	110.7 (85–142)	137.2 (114–155)
Adults	22.4 (19–42)	24/6	n/a	n/a	n/a	n/a

Note: TOWRE = Test of Word Reading Efficiency; BPVS = British Picture Vocabulary Scale.

We assessed the reading proficiency and receptive vocabulary of the child participants only. Reading proficiency was measured by the Test of Word Reading Efficiency–Second Edition (TOWRE; Torgesen et al., 2012). TOWRE is a test of reading fluency that measures how many words (e.g., *book, fine, contain*) and nonwords (e.g., *bave, sline, depate*) pupils can read aloud accurately in 45 s. Raw scores on these two subcomponents of the test were added together to provide a single measure of reading proficiency. Receptive vocabulary was assessed with the British Picture Vocabulary Scale III (BPVS; Dunn & Dunn, 2009), a test in which children are asked to identify the meaning of a spoken word from four pictures.

The study was approved by Royal Holloway’s Research Ethics Committee (Reference No. 3484). Children tested in schools participated on a parental opt-out basis and were asked on multiple occasions throughout testing to provide positive verbal assent. Participants tested in the laboratory provided written consent (or parental consent). Adults were paid for their time, and children received a gift.

### Materials

The materials included four short video clips from animated films for children (see Table 2). These video clips were chosen to have adequate and diverse language content, to be engaging for the full range of primary-school children, and to be unfamiliar to children. The film *Taking Flight* was a “short” produced by an independent animation studio (Moonbot Studios) about a boy playing with his grandfather. The other three clips were from mainstream films that had a U (suitable for all) certification for the United Kingdom on IMDb and Common Sense Media ratings of 6+ (*Rescuers Down Under; The Wild*) and 7+ (*The Road to El Dorado*). The study was approved by the Executive Headteacher of the multischool consortium, and the video clips were considered appropriate for all children in the schools by the consortium’s Lead Practitioner for Early Literacy.

**Table 2.** Characteristics of the Video Clips and Subtitles

Video	Duration (min:sec)	Number of subtitles	Number of words (% of tokens that are unique words)	Average word length (*SD*)	Average word frequency, per million (*SD*)	Flesch Reading Ease score^1^
*Taking Flight* (2016)	5:05	64	283 (61%)	4.0 (1.7)	6,730 (9,953)	87.4
*The Road to El Dorado* (2000)	4:43	86	424 (45%)	3.8 (1.8)	7,536 (10,488)	89.5
*Rescuers Down Under* (1990)	4:21	99	489 (52%)	4.2 (1.9)	6,371 (9,608)	81.2
*The Wild* (2006)	4:13	116	547 (45%)	4.1 (1.9)	7,055 (10,199)	84

It was important for our study to mimic the conditions that children would face if subtitles were switched on by default (as proposed by the Turn on the Subtitles campaign). Thus, the presentation of subtitles in our stimuli broadly followed the BBC Subtitle Guidelines (BBC, 2023). Subtitles were presented as part of the video in one line with an average length of 20 characters (range 3–39). The appearance of each subtitle line was synchronized with the audio. Each word in a subtitle line was on screen for 0.50 s per word on average (range = 0.23–5.50 s). The presentation speed of the subtitles was on average 14 characters per second (*SD =* 6.23; range 1–39). We ensured that the gap between the subtitles was at least 1 s, to avoid a jerky effect.

For all words in the subtitles, we calculated word length in characters and word frequency using the BBC children’s television channel CBBC subcorpus of the SUBTLEX-UK corpus (van Heuven et al., 2014). The words were on average four letters long (*SD =* 1.83; range 1–14) and had an average frequency of 6,926 occurrences per 1 million words (*SD =* 10,069; range 0.007–37,617).

To investigate whether the linguistic content of our video clips was similar to the television programs that primary-school children watch, we compared the words in our video clips to those in five randomly chosen episodes of each of four of the most popular programs on the CBBC: *Danger Mouse*, *The Deep*, *Horrible Histories*, and *The Dumping Ground*. The words in these 20 programs were of similar length (*M =* 4.2 letters, *SD =* 2.1) and frequency (*M* = 6,966 per million, *SD* = 10,076) to those in our video clips (see Table S2 in the Supplemental Material for a full analysis).

To investigate whether the linguistic content of our video clips was accessible to our youngest readers, we compared the words in our video clips to two text benchmarks. The first consisted of the four Level 1 books from the Little Wandle Reading Fluency Scheme designed for use immediately after the phonics screening test in Year 1 (*Blaise and Flint*, *Cycling in Summer*, *Poetry Is Not for Me*, *Talk to the Tail*); the Little Wandle program is one of the most widely used in England. The second consisted of three fiction books from the mandatory reading curriculum in the specific schools attended by our participants: two books used in the summer term of Year 1 (*Giraffes Can’t Dance*, *Esio Trot*) and one used in the autumn term of Year 2 (*The Rainbow Fish*). The words in both of these text sources were of similar length (Wandle: *M* = 4.1 letters, *SD* = 1.90; fiction books: *M =* 4.1 letters, *SD* = 1.90) and frequency (Wandle: *M* = 6,964 per million, *SD* = 10,710; fiction books: *M* = 6,171 per million, *SD* = 9,826) to those in our video clips (see Table S2 for a full analysis).

After each video, we assessed comprehension of the videos via cued recall questions. Following Linebarger (2001), we asked the participants to describe one of the main characters; to describe a critical element of the story; to give the main idea of the story; and to answer a question about some incidental content of the story. The final score ranged from 0 to 20 points per video (with 0 to 5 points per question).

### Apparatus

Participants’ eye movements were recorded by an EyeLink Portable Duo eye tracker (SR Research Ltd, Kanata, Ontario, Canada) with a sampling rate of 1000 Hz. Stimuli were displayed on a MSi Katana GF76 11UD laptop with a screen resolution of 1,920 × 1,080 pixels and a 144-Hz refresh rate. The video resolution was the same as the screen resolution, and the subtitles were part of the video. The subtitles appeared in white 28-point Courier New font against a black background, 82 mm below the center of the screen. They occupied a height of 7% to 8% of the active video height, following the BBC Subtitle Guidelines (BBC, 2023). Each letter of the subtitles took ~0.4° of visual angle, with screen dimensions of 384 × 217 mm and an eye-to-screen distance of 670 mm. A chin rest was used to minimize head movements. Viewing was binocular, but only the right eye was tracked.

### Procedure

Children were tested individually in schools in an unused classroom (*N =* 178) or in the laboratory at Royal Holloway, University of London (*N =* 2). The eye-tracking experiment started with a nine-point calibration and validation process that was repeated until an average precision of 0.5° of visual angle was achieved. Participants were instructed to watch videos with and without subtitles and to answer questions about the content of each video. The presentation of each video started with a drift correction at the center of the screen. Following each video, participants were asked the four comprehension questions. Behavioral testing started with the TOWRE, and this was followed by the BPVS.

Adults were tested individually in the laboratory at Royal Holloway, University of London. The procedure included only the eye-tracking experiment, which was identical to that performed with children. The testing session lasted 50 min on average for children, including breaks, and 30 min for adults.

### Analysis protocol

Our analyses looked first at children’s eye-movement behavior as a function of their school year, reading proficiency, and vocabulary scores. We then compared the eye-movement behavior of children in Year 6 to that of adults.

To analyze participants’ global eye-movement behavior in videos with and without subtitles, we divided the screen into the subtitle region and the main-scene region above the subtitles (the exact parameters of these regions are reported in the Supplemental Material; see Fig. S1). We report the following eye-movement measures for these two regions: *total number of fixations* (fixation count in a region during a given subtitle); *total fixation duration* (the sum of all fixation durations in a region during a given subtitle); and the *number of crossovers* (those saccades that move from the subtitle region to the main scene, and vice versa). For the subtitle condition, we also calculated the *proportion of skipped subtitles* (subtitles without fixations).

To assess whether the eye movements in the subtitle region reflect linguistic processing, we examined participants’ word-based eye-movement behavior. Specifically, we analyzed whether participants were sensitive to word length and frequency during subtitle reading. Following Joseph et al. (2013), Pagán et al. (2020), and Liao et al. (2021, 2022), we report two eye-movement measures: *gaze duration* (the sum of all fixation durations on a word before eyes leave the word) and *total fixation duration* (the sum of all fixation durations on a word, including any refixations). We also compared *mean fixation duration per word* (the average duration of a fixation on a word), *the number of fixations per word*, and *the probability of skipping* a word across different year groups, as previous studies of static text reading in children have shown that these eye-movement measures change with school year (see Blythe & Joseph, 2011, for a review).

Data-cleaning practices vary considerably in the eye-movement literature and can have an impact on the pattern of effects observed (Eskenazi, 2024). Moreover, our paradigm was very different from typical experiments on text reading because of the dynamic text presentation and because of the presence of audio in addition to the subtitles. These differences from static text presentation introduce challenges in determining when fixations may be considered too long or too short. For these reasons, we took a conservative approach and decided not to remove any data before the analyses.

To investigate whether the comprehension of the video content was influenced by the presence of the subtitles, we analyzed participants’ response accuracy in the comprehension task. In this task, each participant could score between 0 and 80 points (20 per video). Before the analysis, to assess the consistency of scoring, we calculated the interrater reliability for comprehension on the basis of 36 evaluations of children’s responses (six randomly selected participants per school year). Each participant’s response was evaluated independently by two trained raters according to the scoring instructions. Both raters were naive about the condition and the exact age of the participants, but they may have been able to guess the approximate age of the participants by their voices, vocabulary, and speech fluency in the audio recordings of the responses. The intraclass correlation coefficient absolute agreement (ICC, type A-1) was calculated using package *irr* (Gamer et al., 2019). The ICC was 0.90, with a 95% confidence interval (CI) of [0.47, 0.96]; Rater 1, mean score = 61%; Rater 2, mean score = 58%, which indicates good interrater reliability, according to the criteria defined by Koo and Li (2016).

Data were analyzed using linear mixed-effects models for continuous outcome variables (e.g., for the number of fixations, fixation durations, and response accuracy) and using logistic mixed-effects models for binary outcome variables (e.g., the probability of making a crossover or the probability of skipping; Baayen et al., 2008). Analyses were conducted with R (R Core Team, 2020). Models were estimated with the *lme4* package (Bates et al., 2015), and tables showing model outcomes were created with the *sjPlot* package (Lüdecke, 2017). For the fixed-effects structure, condition was coded with sum contrasts (1 referred to the subtitle condition and −1 to the no-subtitle condition), each continuous variable was centered on the grand mean of the continuous variable, and word frequency was log-transformed (0.1 was substituted for N/A). To compare children in Year 6 with adults, we used sum contrasts (1 referred to adults and −1 to Year 6 students). All models included random intercepts for participants, items (subtitles, words, or videos), and participants’ school, as well as all possible random slopes. We started with the maximal model and optimized its random structure so that it was best supported by both the experimental design and the data (Bates et al., 2018).

Year group, reading proficiency, and receptive vocabulary scores were correlated; thus, we quantified the severity of multicollinearity by estimating the variance inflation factors (VIF) using the *vif()* function in the *car* package. This process led to the exclusion of BPVS from all analyses (positive Spearman correlation with school year: *r* = .76, *p* < .001). Following exclusion of BPVS, the VIF in all models was below 4, indicating no remaining multicollinearity problems. School year and TOWRE scores were also correlated (positive Spearman correlation: *r* = .63, *p* < .001); however, we included both measures in the analysis because TOWRE was a direct measure of reading proficiency that, according to our hypothesis, should be related to the attention to subtitles, and school year was an indirect measure of reading skills that was an intrinsic part of our experimental design. We chose to retain TOWRE instead of BPVS because it is a measure of reading (as opposed to vocabulary), but following the editor’s suggestion, we repeated the analyses with BPVS.

To analyze significant interaction effects, we used the *sim_slopes()* function and the *contrast()* function in the R packages *interactions* and *emmeans* (Lenth et al., 2021), which contrasted the slopes of the predictors in the model or fitted the models with nested contrasts.

Because of the way that variance is partitioned in mixed-effects models (e.g., Rights & Sterba, 2019), there is no consensus on how to calculate standardized effect sizes like Cohen’s *d* for main effects or interactions. We therefore report unstandardized effect sizes in the form of βs and odds ratios (*OR*s), as well as data means and 95% CIs. Beta (β) is the fixed-effect coefficient (logit-transformed for logistic models), which refers to the estimated difference between conditions after we controlled for random effects. For example, for the fixed effect of condition, where 1 refers to the subtitle condition and −1 to the no-subtitle condition, β = 1.23 would mean that the participants made 2.46 more fixations in a region in the subtitle condition compared with the no-subtitle condition. *OR* (derived from β) measures the difference in odds of an event occurring (vs. not occurring) in one level of a fixed effect compared with another. For example, for the fixed effect of school year, *OR* denotes the change in odds for the event to occur with each advancing school year (e.g., *OR* < 1 would mean that the odds of a subtitle being skipped decreases with school year).

## Results

During the debriefing, we asked the child participants whether they had seen any of the films before. Six children indicated that the characters in *The Road to El Dorado* and *Rescuers Down Under* seemed familiar, but they did not remember any details of the films. We decided that this level of familiarity was not sufficient to warrant removal of these participants.

### Global analyses of the subtitle region

**Fig. 1.**
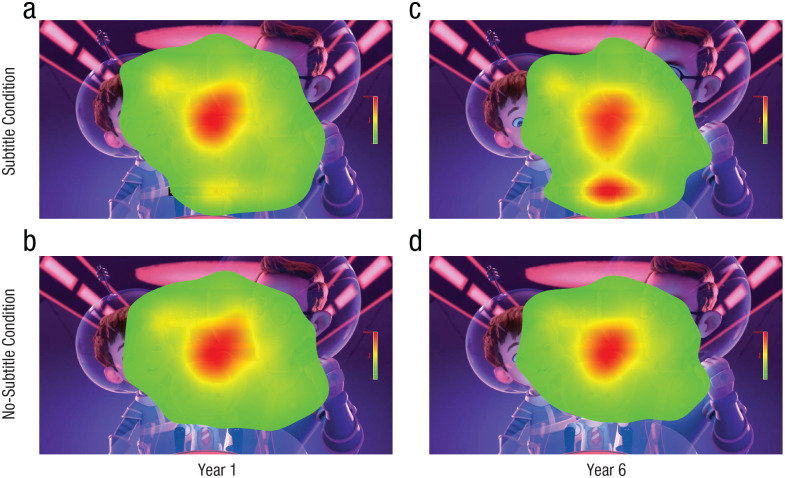
Heat maps created from fixations aggregated across all participants who watched the video *Taking Flight*. In (a), we show children in Year 1 viewing a video with subtitles; in (b), children in Year 1 viewing a video without subtitles; in (c), children in Year 6 viewing a video with subtitles; and in (d), children in Year 6 viewing a video without subtitles. The color varies from green (shorter fixation durations) to red (longer fixation durations) and illustrates the density of fixations in a particular region of the screen.

The first series of analyses aimed to investigate whether attention to the subtitle region was influenced by the presence of subtitles on the screen and whether this effect was modulated by the children’s school year and level of reading proficiency. Specifically, we analyzed the effect of condition (subtitle versus no-subtitle) and its interaction with school year and TOWRE on various eye-movement measures. We provide an illustration of global eye-movement behavior in Figure 1 and in the form of two videos that aggregate eye movements for pupils in Year 1 and Year 6 watching a 32-s clip of *Taking Flight*. The first video compares the eye movements of children in Year 1 and Year 6 watching the video with subtitles, whereas the second video compares the eye-movements of children in Year 6 watching the video with and without subtitles (see the Open Science Framework site for this project: https://osf.io/uk8bt/).

#### Fixations

**Fig. 2.**
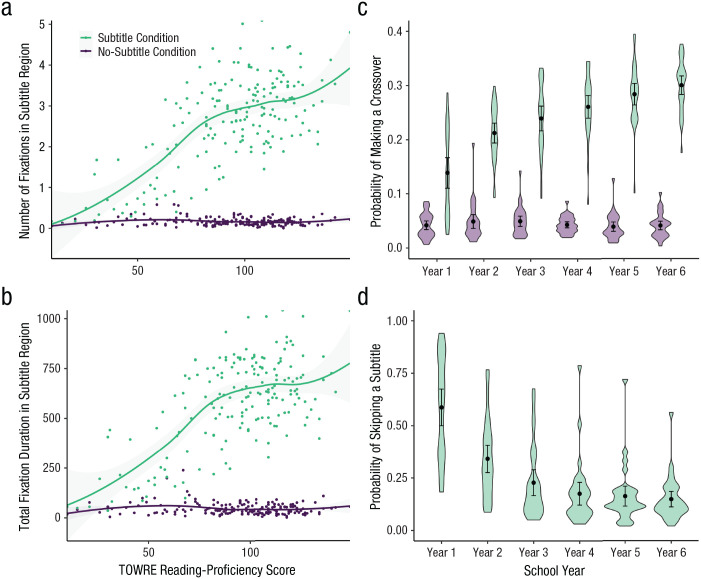
Eye movements in the global analysis. In (a) is shown the interaction between condition (with subtitles, green line; without subtitles, purple line) and Test of Word Reading Efficiency (TOWRE) on the total number of fixations in the subtitle region. In (b) is shown the interaction between condition and TOWRE on total fixation duration in the subtitle region. In (c) is illustrated the probability of making a saccade between the subtitle and the main-scene regions and vice versa (a crossover) across six year groups. In (d) is shown the probability of skipping a subtitle across six year groups. Error bars represent 95% confidence intervals.

Children made more fixations to the subtitle region (β = 1.23, 95% CI = [1.22, 1.24]; *t* = 218.23; *p* < .001) and spent more time looking at the subtitle region (β = 265.29, 95% CI = [262.66, 267.92], *t* = 197.66, *p* < .001) in the subtitle condition compared with the no-subtitle condition (see Table 3 for a summary of results). These results held for all year groups (see Table S3 for model outputs). However, we found condition-by-TOWRE interactions for both of these dependent variables (β = 0.01, 95% CI = [0.01, 0.01], *t* = 48.08, *p* < .001 for the number of fixations; β = 2.59, 95% CI = [2.46, 2.72], *t* = 39.97, *p* < .001 for the total fixation duration), indicating that the effect of condition was greater for children with higher TOWRE scores (see Figs. 2a and 2b). Model outputs and further statistical analysis of the interaction are presented in Table S4 in the Supplemental Material.

**Table 3.** Means and 95% Confidence Intervals of Global Eye-Movement Measures per Subtitle in the Two Conditions

School year	Condition	Total number of fixations		Total fixation duration (ms)		Probability of a crossover	Probability of skipping a subtitle
		Subtitles	Main scene	Subtitles	Main scene		
Year 1	subtitle	1.3 [0.9, 1.7]	3.9 [3.6, 4.2]	326 [242, 409]	1216 [1117, 1316]	0.14 [0.11, 0.17]	0.59 [0.50, 0.67]
	no-subtitle	0.2 [0.1, 0.2]	4.5 [4.2, 4.8]	46 [37, 56]	1457 [1406, 1508]	0.04 [0.03, 0.05]	
Year 2	subtitle	2.3 [2.0, 2.7]	3.3 [3.1, 3.6]	535 [466, 604]	954 [876, 1032]	0.21 [0.19, 0.23]	0.34 [0.28, 0.41]
	no-subtitle	0.2 [0.1, 0.2]	4.5 [4.3, 4.8]	50 [35, 66]	1432 [1379, 1485]	0.05 [0.04, 0.06]	
Year 3	subtitle	2.9 [2.6, 3.4]	3.2 [3.0, 3.6]	648 [573, 723]	852 [771, 934]	0.24 [0.22, 0.26]	0.23 [0.17, 0.29]
	no-subtitle	0.2 [0.2, 0.2]	4.7 [4.5, 5.0]	55 [41, 69]	1466 [1410, 1522]	0.05 [0.04, 0.05]	
Year 4	subtitle	3.2 [2.9, 3.4]	3.3 [3.0, 3.5]	671 [603, 740]	824 [752, 896]	0.26 [0.24, 0.28]	0.18 [0.12, 0.23]
	no-subtitle	0.2 [0.1, 0.2]	4.8 [4.6, 5.0]	45 [38, 53]	1469 [1435, 1504]	0.04 [0.03, 0.05]	
Year 5	subtitle	3.1 [2.8, 3.4]	3.2 [2.9, 3.4]	640 [577, 704]	850 [783, 918]	0.28 [0.26, 0.30]	0.16 [0.12, 0.21]
	no-subtitle	0.1 [0.1, 0.2]	4.6 [4.3, 4.8]	41 [32, 49]	1516 [1481, 1550]	0.04 [0.03, 0.05]	
Year 6	subtitle	2.9 [2.6, 3.1]	3.2 [3.0, 3.5]	636 [591, 682]	857 [797, 916]	0.30 [0.28, 0.32]	0.15 [0.11, 0.19]
	no-subtitle	0.1 [0.1, 0.2]	4.6 [4.4, 4.9]	37 [30, 44]	1485 [1447, 1523]	0.04 [0.03, 0.05]	
Adults	subtitle	2.6 [2.3, 2.9]	3.6 [3.3, 3.8]	509 [447, 570]	924 [864, 984]	0.29 [0.27, 0.31]	0.17 [0.13, 0.22]
	no-subtitle	0.1 [0.1, 0.1]	4.8 [4.5, 5.0]	28 [19, 38]	1482 [1437, 1527]	0.02 [0.02, 0.03]	

Note: Confidence intervals are in brackets.

#### Crossovers

Children made more crossovers between the main scene and the subtitle regions in the subtitle condition compared with the no-subtitle condition (β = 0.98, *OR* = 2.65, 95% CI = [0.96, 0.99], *z* = 123.44, *p* < .001). This result held for all year groups (see Table S3 for model outputs). We also found a condition-by-TOWRE interaction (β = 0.004, *OR* = 1.004, 95% CI = [0.003, 0.005], *z* = 10.80, *p* < .001) and a condition-by-year interaction (β = 0.05, *OR* = 1.05, 95% CI = [0.04, 0.06], *z* = 8.62, *p* < .001). These interactions indicate that the effect of condition was greater for children who attained higher TOWRE scores and who were in later school years (see Fig. 2c). Model outputs and further statistical analysis of the interactions are presented in Table S5 in the Supplemental Material.

#### Skipping

For the subtitle condition only, we estimated the probability of skipping a whole subtitle. We found that children with higher TOWRE scores were less likely to skip subtitles than children with lower scores (β = −0.04, *OR* = 0.96, 95% CI = [−0.04, −0.03], *z* = −9.43, *p* < .001). Likewise, children in later school years were less likely to skip subtitles than children in earlier school years (β = −0.22, *OR* = 0.80, 95% CI = [−0.34, −0.10], *z* = −3.54, *p* < .001); see Figure 2d. Model outputs are presented in Table S6 in the Supplemental Material.

#### Comparison between years

We conducted an exploratory analysis comparing global eye-movement behavior across year groups. Results showed that pupils in the subtitle condition in Year 2 made more fixations (β = 1.01, 95% CI = [0.78, 1.24], *t* = 8.65, *p* < .001), spent more time looking at the subtitle region (β = 207.69, 95% CI = [158.01, 257.37], *t* = 8.19, *p* < .001), were more likely to make crossovers between the regions (β = 0.50, *OR* = 1.66, 95% CI = [0.46, 0.55], *z* = 20.35, *p* < .001), and were less likely to skip subtitles (β = −1.58, *OR* = 0.21, 95% CI = [−2.18, −0.98], *z* = −5.14, *p* < .001) compared with pupils in Year 1. Similarly, pupils in the subtitle condition in Year 3 made more fixations (β = 0.68, 95% CI = [0.45, 0.91], *t* = 5.81, *p* < .001), spent more time looking at the subtitle region (β = 114.20, 95% CI = [64.51, 163.88], *t* = 4.51, *p* < .001), were more likely to make crossovers between the regions (β = 0.12, *OR* = 1.13, 95% CI = [0.08, 0.16], *z* = 5.81, *p* < .001), and were less likely to skip subtitles (β = −0.88, *OR* = 0.42, 95% CI = [−1.48, −0.27], *z* = −2.85, *p* = .004) compared with pupils in Year 2. No differences were found between the other pairs of years in the number and duration of fixations on subtitles or in the probability of skipping a subtitle. Model outputs are presented in Tables S7 and S8 in the Supplemental Material.

### Word-based analyses of subtitles

The second series of analyses aimed to determine whether children actually read the subtitles when they fixated on them. Effects of length and frequency on fixation duration are typically interpreted as reflecting linguistic processing (Joseph et al., 2009, 2013). Thus, we investigated whether this relationship was present in our data and whether it varied as a function of children’s school year and TOWRE scores. We also investigated whether standard eye-movement measures that reflect the effectiveness of word processing were modulated by children’s school year and reading proficiency.

#### Length and frequency

Longer words had longer gaze durations (β = 0.03, 95% CI = [0.03, 0.04], *t* = 10.22, *p* < .001) and total fixation durations (β = 0.04, 95% CI = [0.04, 0.05], *t* = 10.68, *p* < .001) compared with shorter words. Similarly, more frequent words had shorter gaze durations (β = −0.02, 95% CI = [−0.02, −0.01], *t* = −8.94, *p* < .001) and total fixation durations (β = −0.02, 95% CI = [−0.02, −0.01], *t* = −8.82, *p* < .001) compared with less frequent words. There was an interaction between school year and frequency in the analysis of both gaze duration (β = 0.002, 95% CI = [0.0004, 0.003], *t* = 2.49, *p* = .014) and total fixation duration (β = 0.002, 95% CI = [0.001, 0.004], *t* = 3.03, *p* = .002), indicating that the effect of frequency reduces with school year. These findings are consistent with research on static text reading (Joseph et al., 2013; Tiffin-Richards & Schroeder, 2015). However, these interactions should be interpreted with caution because of the large amount of missing data in Year 1 (59% of subtitles were skipped) and Year 2 (34% of subtitles were skipped). No other interactions reached significance. Model outputs are presented in Table S9 in the Supplemental Material.

#### Standard eye-movement measures by year group

Results showed that children in later school years and that children with higher TOWRE scores had shorter fixation durations on words. These effects were observed for mean fixation durations (β = −0.02, 95% CI = [−0.04, −0.003], *t* = −2.30, *p* = .023 for school year; β = −0.002, 95% CI = [−0.004, −0.001], *t* = −4.48, *p* < .001 for TOWRE), gaze durations (β = −0.04, 95% CI = [−0.05, −0.02], *t* = −4.49, *p* < .001 for school year; β = −0.003, 95% CI = [−0.004, −0.002], *t* = −5.09, *p* < .001 for TOWRE), and total fixation durations (β = −0.04, 95% CI = [−0.05, −0.02], *t* = −4.60, *p* < .001 for school year; β = −0.002, 95% CI = [−0.003, −0.001], *t* = −3.86, *p* < .001 for TOWRE). These results are consistent with findings from studies on static text reading (Blythe & Joseph, 2011). The number of fixations per word also decreased as school year increased (β = −0.03, 95% CI = [−0.05, −0.02], *t* = −3.91, *p* < .001), as observed in static text reading (Blythe & Joseph, 2011). Finally, for subtitles that were not skipped entirely, the probability of skipping a word in first-pass reading decreased with higher TOWRE scores (β = −0.01, *OR* = 0.99, 95% CI = [−0.01, −0.01], *z* = −28.57, *p* < .001). This effect is unlike that observed in static text reading (Blythe & Joseph, 2011). Exploratory analyses (see Table S10) suggested that the initial words of the subtitle are unlikely to be fixated in the first pass, but that superior readers are more likely to fixate on these words than poorer readers. Further saccadic targeting research will be necessary to understand how readers interrogate word-level information in subtitles and how this varies with reading proficiency. Model outputs are presented in Tables S9 and 10 in the Supplemental Material. Sample means and confidence intervals for the five measures are reported in Table 4.

**Table 4.** Means and 95% Confidence Intervals of Five Word-Based Eye-Movement Measures in the Subtitle Region in Children and Adults

School year	Gaze duration (ms)	Total fixation duration (ms)	Mean fixation duration (ms)	Number of fixations	Probability of skipping
Year 1	403 [393, 413]	465 [454, 477]	299 [291, 306]	1.62 [1.59, 1.65]	0.66 [0.65, 0.67]
Year 2	336 [331, 342]	388 [382, 395]	266 [262, 270]	1.52 [1.50, 1.53]	0.58 [0.57, 0.59]
Year 3	294 [290, 298]	350 [345, 355]	245 [242, 248]	1.46 [1.45, 1.48]	0.51 [0.50, 0.51]
Year 4	282 [278, 285]	338 [333, 342]	241 [238, 244]	1.43 [1.42, 1.45]	0.51 [0.50, 0.52]
Year 5	267 [263, 271]	318 [313, 322]	234 [231, 238]	1.39 [1.38, 1.40]	0.50 [0.50, 0.51]
Year 6	293 [287, 299]	342 [335, 349]	260 [254, 266]	1.37 [1.35, 1.38]	0.55 [0.54, 0.55]
Adults	224 [221, 227]	262 [258, 266]	202 [200, 205]	1.32 [1.31, 1.33]	0.53 [0.52, 0.54]

Note: The number of fixations was calculated for the words that were fixated at least once. The probability of skipping words was derived for the subtitles that were not skipped entirely. Confidence intervals are in brackets.

### Comprehension

The analysis of comprehension tested whether subtitles influenced understanding of the videos and whether this effect was modulated by children’s school year and reading proficiency.

The mean response accuracy in comprehension questions ranged from 11% to 98% by participant and from 70% to 81% by video. Accuracy increased with school year: 57% in Year 1 (55% in the subtitle condition and 59% in the no-subtitle condition); 72% in Year 2 (75% and 69%, respectively); 80% in Year 3 (81% and 78%, respectively); 82% in Year 4 (82% and 82%, respectively); 85% in Year 5 (85% and 84%, respectively); and 86% in Year 6 (86% and 86%, respectively). Statistical analysis showed no effect of subtitle condition on comprehension (β = 0.08, 95% CI = [−0.08, 0.25], *t* = 0.97, *p* = .334); however, there was a condition-by-TOWRE interaction just under the significance threshold (β = 0.01, 95% CI = [0.00, 0.02], *t* = 2.16, *p* = .032) indicating that the relationship between reading proficiency and comprehension was slightly greater in the subtitle condition (β = 0.04, 95% CI = [0.03, 0.06], *t =* 5.30, *p* < .001) than in the no-subtitle condition (β = 0.03, 95% CI = [0.01, 0.04], *t* = 3.10, *p* < .001). Model outputs are presented in Table S11 in the Supplemental Material.

We conducted an exploratory correlational analysis to test whether pupils who made more fixations and longer fixations on subtitles had higher comprehension accuracy. However, we found no evidence for this once the effect of age was taken into account (see Table S12 in the Supplemental Material).

### Further results reported in the supplemental material

#### Comparison between Year 6 and adults

Children in Year 6 spent more time looking at the subtitles and at individual words within the subtitles than adults did; they also showed poorer comprehension. There were no differences between the groups in the effects of length and frequency on eye-movement measures. Detailed results are reported in Appendix S1 and Tables S13 to S18 in the Supplemental Material.

#### Processing of subtitles for children in Year 1

Children in Year 1 skipped 59% of whole subtitles, and in the subtitles that they did fixate on, they skipped 66% of the words. Exploratory analyses showed that these children were more likely to fixate on subtitles that were longer (in length or duration) and that they were more likely to fixate on words positioned toward the end of the subtitle rather than toward the beginning. Qualitative evidence suggests that these children tended to fixate more on subtitles during periods of dialogue than periods of intense action. Detailed analysis is reported in Appendix S2 in the Supplemental Material.

#### Analyses using vocabulary scores (rather than reading scores) as predictors

We refitted all models from the analyses reported above, substituting TOWRE with BPVS. The results from the models with BPVS were broadly consistent with those using TOWRE. Model outputs are presented in Tables S19 to S24 in the Supplemental Material.

## Discussion

Our main finding was that children’s reading proficiency was associated with the extent to which they looked at subtitles. Critically, children in Year 1 ignored 59% of whole subtitles and skipped 66% of the words in the remaining subtitles. Most of these children would have had almost 2 years of reading instruction at the point of testing, and our analysis of the characteristics of words used in our subtitles indicated that they were similar to those used in curricular reading materials for these children. Every child in our Year 1 sample had passed the national phonics screening test (average score 38/40 items), indicating outstanding foundational decoding skills. We speculate that these children showed low engagement with the subtitles because they did not have sufficient reading fluency to cope with the dynamic and time-limited nature of the subtitles.

Our results suggest that the potential impact of same-language subtitles on improving reading skills may be quite limited in the case of younger readers. However, our findings also showed a progressive increase across Years 2 and 3 in children’s attention to subtitles. By the end of Year 3, the children in our sample spent substantial time looking at subtitles, and their behavior could not be distinguished from that of older children. These data suggest that although same-language subtitles may not support the earliest stages of learning to read, they could be a useful tool for building reading fluency and gaining print exposure in those children who have already achieved sufficient reading proficiency to engage with subtitles.

We did not find an impact of subtitles on comprehension. Previous research has indicated that subtitles may impair comprehension in young children (Linebarger, 2001). The fact that we did not replicate this disadvantage in a larger sample is important because it dispels concerns that default subtitling may cause harm. We did not anticipate a benefit of subtitles on comprehension, because for emerging readers watching videos in their native language, spoken-language processing should be easier than reading. One interesting question is why older children and adults spend so much time attending to subtitles when they provide no obvious benefits for understanding. These data may be a reflection of the automaticity of skilled reading (d’Ydewalle et al., 1991): It is difficult for proficient readers to ignore printed words (Stroop, 1935). These data may also indicate that subtitles yield other benefits for proficient readers; qualitative debriefing of adult participants suggested that they use subtitles to minimize comprehension effort, understand accents, and permit multitasking. That said, we acknowledge that our measure of comprehension (based on Linebarger, 2001) may not have been sensitive enough to detect any benefit of subtitles in the more proficient readers.

Our word-based analyses suggest that when children were looking at the subtitles they were reading them. This inference is based on the observation that children showed longer fixation durations for longer and less frequent words. Likewise, we observed typical developmental patterns on fixation durations: Fixation durations decrease as reading proficiency increases. However, our word-skipping data indicate that reading subtitles is not like reading static text. Even when considering only those subtitles that had at least one fixation, the word-skipping rates for all year groups were very high (≥ 50% in the first pass), with greater skipping in the less proficient readers. Word skipping is considered an indicator of reading efficiency, but the dynamic nature of subtitles means that readers rarely start reading from the first words on the subtitles, and less proficient readers may be forced to skip many words. Furthermore, even for more proficient readers, engaging in subtitle reading necessarily involves cross-modal processing and divided attention (Liao et al., 2022). Further research will be necessary to understand how viewers combine different sources of information (video, soundtrack, and subtitles) to optimize comprehension and how this is reflected in their subtitle-reading behavior.

This study offers the first evidence that children read same-language subtitles while watching videos in their native language. However, we caution that our sample excluded pupils with special educational needs and those with English as an additional language and may therefore have consisted of pupils with higher-than-average reading proficiency. It is possible that children with weaker reading skills would be less engaged with the subtitles than the pupils we observed in this study. Likewise, we acknowledge that our analyses of the relationship between reading proficiency and subtitle viewing were based on observational data, making it difficult to determine that reading proficiency plays a definitive causal role in subtitle-viewing behavior (Rohrer, 2018). It may be that older children have more experience with subtitles than younger children and that this natural confound in our experiment was behind the apparent effects of reading proficiency. It is not possible to determine from our study how prior experience with subtitles influences fixation behavior beyond reading fluency.

The critical conclusion from this study is that a certain degree of reading proficiency may be a prerequisite for effective engagement with same-language subtitles. Our study suggests that most English-speaking children are unlikely to have sufficient reading skill after 2 years of classroom instruction to engage substantively with the same-language subtitles used in programs typical for this age group. However, once children are able to read sufficiently quickly (often by the third or fourth year of reading instruction, for typically developing children), they are likely to pay considerable attention to the subtitles, opening the possibility that subtitles could provide some of the vital reading practice necessary for children to become skilled readers. Intervention studies will be needed to discover whether same-language subtitles play a causal role in the development of reading skill.

## Supplemental Material

sj-docx-1-pss-10.1177_09567976251325789 – Supplemental material for Where Do Children Look When Watching Videos With Same-Language Subtitles? Supplemental material, sj-docx-1-pss-10.1177_09567976251325789 for Where Do Children Look When Watching Videos With Same-Language Subtitles? by Anastasiya Lopukhina, Walter J. B. van Heuven, Rebecca Crowley and Kathleen Rastle in Psychological Science

## Footnotes

1. Flesch Reading Ease scores were included to demonstrate that the subtitles across the four videos were of similar reading difficulty. These scores should not be used to benchmark subtitle difficulty to U.S. grade-level equivalents using calculators available on the Internet. These mappings come from Flesch (1949, p. 149) and are not based on substantive testing of children’s reading. Moreover, these mappings do not go below U.S. 5th grade, as Flesch (1948, p. 225) considered pupils who had completed 4th grade as “barely functionally literate.”

## Transparency

*Action Editor:* Louis Moses

*Editor:* Simine Vazire

*Author Contributions*

**Anastasiya Lopukhina:** Conceptualization, Data curation, Formal analysis, Investigation, Methodology, Writing – original draft, Writing – review & editing.

**Walter J. B. van Heuven:** Conceptualization, Funding acquisition, Methodology, Writing – review & editing.

**Rebecca Crowley:** Investigation, Writing – review & editing.

**Kathleen Rastle:** Conceptualization, Funding acquisition, Methodology, Writing – original draft, Writing – review & editing.

**Artificial Intelligence:** No artificial-intelligence-assisted technologies were used in this research or the creation of this article.

**Ethics:** This research complies with the Declaration of Helsinki (2023) and received approval from a local ethics board (Reference No. 3484).

**Open Practices:** Study disclosures. Preregistered: The hypotheses, methods, and analysis plan were preregistered (https://doi.org/10.17605/OSF.IO/NVXFM). There were minor deviations from the preregistration (for details, see Table S1 in the Supplemental Material). Materials: Some materials are publicly available (https://doi.org/10.17605/OSF.IO/PQYWF). The videos cannot be shared publicly because they are copyright protected; however, they can be made available upon request by contacting Kathleen Rastle (kathy.rastle@rhul.ac.uk). We also report the titles of the movies and the time stamps of the episodes and share the subtitles so that the exact episodes can be identified. Data: All raw data are publicly available (https://doi.org/10.17605/OSF.IO/5TD87). Analysis scripts: All analysis scripts are publicly available (https://doi.org/10.17605/OSF.IO/5BDAK). Computational reproducibility: The computational reproducibility of the results has been independently confirmed by the journal’s STAR team.
