# Supplementary material for: Where Do Children Look When Watching Videos With Same-Language Subtitles?
Source: Psychol Sci. 2025 Apr 2;36(4):223–36. doi: 10.1177/09567976251325789 (PMC13428831; doi:10.1177/09567976251325789)
Supplement: sj-docx-1-pss-10.1177_09567976251325789 – Supplemental material for Where Do Children Look When Watching Videos With Same-Language Subtitles? [file sj-docx-1-pss-10.1177_09567976251325789.docx]

**Appendix S1**

***Comparison with adults***

This analysis aimed to explore whether viewing behavior while watching videos with same-language subtitles in children from Year 6 was comparable with viewing behavior in skilled adult readers.

**Global analysis**. The analyses of eye movements in the subtitle region showed condition-by-group interactions (*β* = -0.06; 95% CI = [-0.07, -0.04]; *t* = -7.56; *p* < .001 for the number of fixation; *β* = -29.70; 95% CI = [-33.42, -25.98]; *t* = -15.64; *p* < .001 for the total fixation duration), indicating that children in Year 6 made more fixations (*β* = 0.26; 95% CI = [0.04, 0.48]; *z* = 2.97; *p* = .01) and had longer total fixation duration on subtitles (*β* = 125.85; 95% CI = [74.10, 177.60]; *z* = 6.07; *p* < .001) compared to adults but only in the subtitle condition. We also found a condition-by-group interaction for the number of crossovers (*β* = 0.16; *OR* = 1.17; 95% CI = [0.08, 0.24]; *z* = 3.91; *p* < .001), indicating that children in Year 6 were more likely to make a crossover between the subtitle and the main scene regions (*β* = 0.68; 95% CI = [0.25, 1.11]; *z* = 3.91; *p* < .001) compared to adults, but only in the no-subtitle condition. Finally, adults and children skipped similar number of whole subtitles (*β* = 0.08; *OR* = 1.08; 95% CI = [-0.19, 0.34]; *z* = 0.59; *p* = .56). Model outputs are presented in Tables S13-15.

**Word-based analysis of subtitles**. The analysis of length and frequency effects showed that longer words required longer gaze duration (*β* = 0.03; 95% CI = [0.02, 0.04]; *t* = 7.35; *p* < .001) and total fixation duration (*β* = 0.04; 95% CI = [0.03, 0.05]; *t* = 8.52; *p* < .001) compared to shorter words. More frequent words required shorter gaze duration (*β* = -0.01; 95% CI = [-0.01, -0.01]; *t* = -6.68; *p* < .001) and total fixation duration (*β* = -0.01; 95% CI = [-0.02, -0.01]; *t* = -6.40; *p* < .001) compared to less frequent words. There were no group-by-length or group-by-frequency interactions. Overall, adults had shorter mean fixation duration (*β* = -0.12; 95% CI = [-0.16, -0.08]; *t* = -5.62; *p* < .001), gaze duration (*β* = -0.13; 95% CI = [-0.18, -0.09]; *t* = -6.00; *p* < .001), and total fixation duration (*β* = -0.14; 95% CI = [-0.18, -0.09]; *t* = -6.20; *p* < .001) and they made fewer fixations (*β* = -0.03; 95% CI = [-0.06, -0.00]; *t* = -2.06; *p* = .04) on words compared to children in Year 6. Both groups skipped comparable number of words in subtitles (*β* = -0.01; *OR* = 0.99; 95% CI = [-0.10, 0.07]; *z* = -0.30; *p* = .76). Detailed summaries of model fits are reported in Tables S16-17.

**Comprehension**. The mean response accuracy for adults (93%; 93% in the subtitle and 93% in the no-subtitle condition) was higher than for Year 6 children (86%), (*β* = 0.70; 95% CI = [0.39, 1.02]; *t* = 4.39; *p* < .001). We found no effect of condition or a condition-by-group interaction. Model outputs are presented in Table S18.

**Appendix S2**

***Eye movements in videos with subtitles in Year 1***

The global analysis showed that in the subtitle condition, children in Year 1 spent less time looking at the subtitle region, were less likely to make a crossover between the regions compared to children in Year 2 and skipped 59% of whole subtitles. Their viewing behavior stood out compared to that of children from the other school years. To understand this viewing behavior in Year 1, we analyzed the skipping of subtitles and the distribution of fixations when children were looking at the subtitles.

**Skipped subtitles**. The proportion of whole subtitles skipped by participants ranged from 0.18 to 0.94 (*M* = 0.59; 95% *CI* = 0.50-0.67). Year 1 children with higher TOWRE scores skipped fewer whole subtitles (negative Pearson correlation: *r* = -0.67, *p* < .001). We also found that some subtitles were particularly likely to be skipped (≥ 0.80; *N* = 97 subtitles), whereas others were rarely skipped (≤ 0.20; *N* = 29 subtitles). Most of the subtitles that were rarely skipped were from *Taking Flight* and *The Wild* videos. These were dialogues between the characters with little action in the video, e.g. *You know, that wagon used to be your dad's. What say we take it out for a spin, huh?* (from *Taking Flight*, the grandfather talks to his grandson); *What's he gonna think of me when he finds out? – I don't know.* (from *The Wild*, the father lion talks with his friend).

The subtitles that were frequently skipped were mostly from *The Road to El Dorado* and *Rescuers Down Under* videos (79 out of 97 subtitles). When these subtitles were on screen, there was a lot of action or unexpected events in the video, e.g. *Yeah. We're at least in a rowboat. – We're in a rowboat. Exactly. We've missed nothing.* (from *The Road to El Dorado*, the characters were talking while sharks started following the boat). Overall, it seems that children in Year 1 did not look at the subtitles when the content of the video required or engaged more attention.

Children in Year 1 were less likely to skip longer subtitles compared to shorter subtitles (*β* = -0.04; *OR* = 0.96; 95% CI = [-0.07, -0.01]; *z* = -2.93; *p* = .003). For example, they skipped one-word subtitles in 64% of cases and ten-word subtitles in 45% of cases. Longer subtitles were probably more salient and attracted more attention than shorter subtitles. Also, the children were less likely to skip subtitles that stayed on screen for a longer time (*β* = -0.0001; *OR* = 0.99; 95% CI = [-0.0002, -0.00003]; *z* = -2.95; *p* = .003).

**Subtitles with fixations**. When children in Year 1 fixated on the subtitles, they made more fixations on longer subtitles compared to shorter subtitles (*β* = 0.13; 95% CI = [0.08, 0.18]; *t* = 4.93; *p* < .001) and these fixations were longer as reflected in the total fixation duration (*β* = 30.92; 95% CI = [16.63, 45.21]; *t* = 4.24; *p* < .001). Similarly, the subtitles that were present on the screen for a longer time received more fixations (*β* = 0.0005; 95% CI = [0.0004, 0.0006]; *t* = 7.28; *p* < .001) and had longer total fixation durations (*β* = 0.17; 95% CI = [0.14, 0.21]; *t* = 9.18; *p* < .001) compared to the subtitles that were present for a shorter time. In combination with the outcomes of the word-based analysis, these results suggest that when children in Year 1 looked at the subtitles they were reading them.

Finally, for the subtitles that were fixated at least once, we analyzed which words were more likely to be skipped in the first pass as a function of their position in a sentence. We found that children were less likely to skip words closer to the end of the subtitle (*β* = -0.13; *OR* = 0.88; 95% CI = [-0.21, -0.04]; *z* = -2.87; *p* = .004). For example, in ten-word sentences, children skipped the first word in 86% of cases and the tenth word in 57% of cases. These data indicate that in multi-word subtitles, children in Year 1 preferred to look at the final words, perhaps because they were more likely to be able to read these synchronously with the audio.


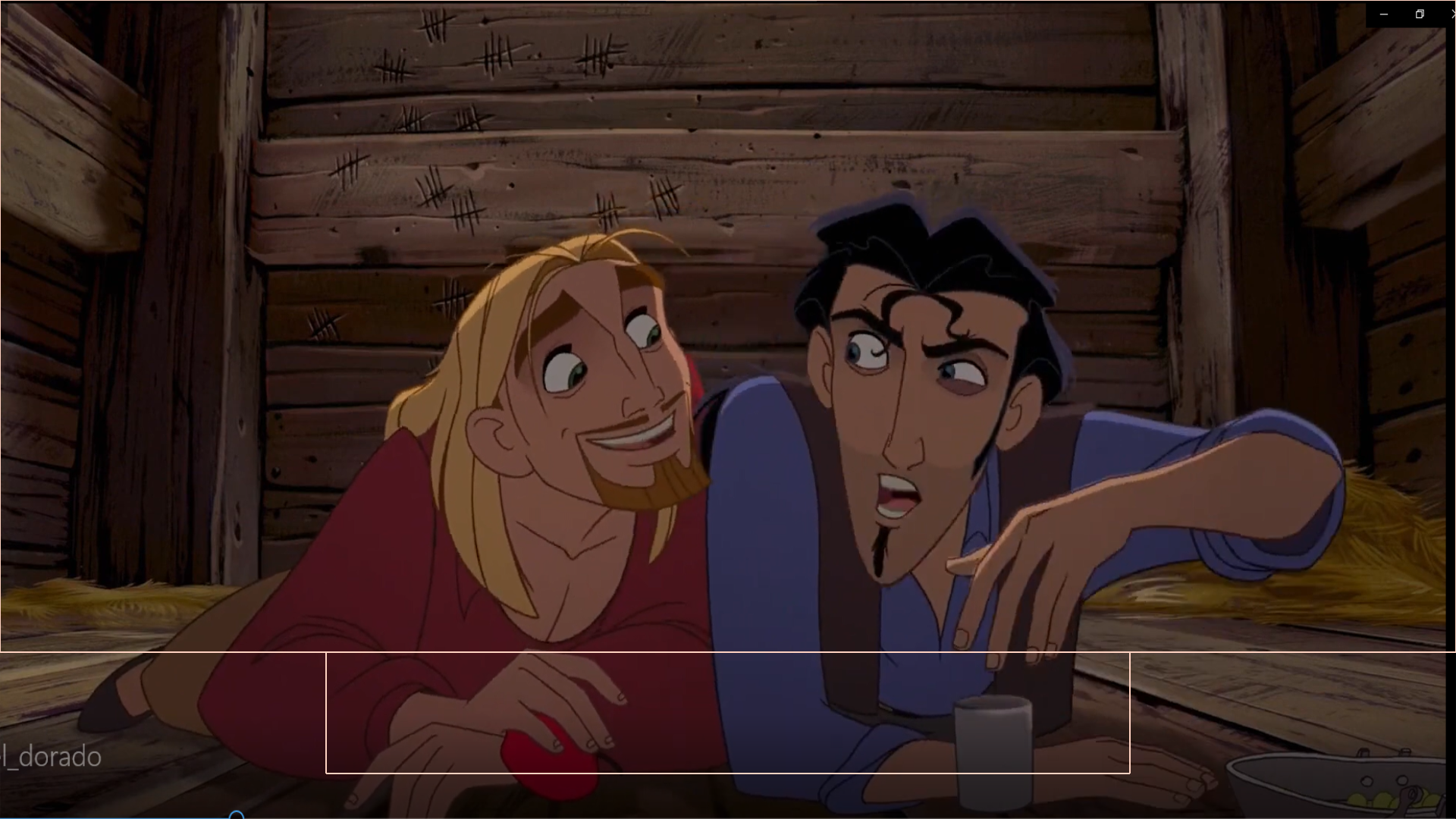


**Fig. S1**. The illustration of the main scene and the subtitle regions in the eye-tracking experiment. The main scene region took an area of 1920 (the whole screen width) x 860 pixels and started from the top of the display screen. The subtitle region took an area of 1060 x 160 pixels and started right below the main scene region. It was symmetrical and started 430 pixels from the left side and ended 430 pixels to the right side of the screen.

Table S1. Preregistration deviations

| **No.** | **Type** | **Original wording** | **Deviation description** |
| --- | --- | --- | --- |
| 1 | Sample size:  Collected more participants than planned | In total, we aim to recruit at least 24 participants per year group but, if possible, we will try to increase this number. | In total, 180 children (30 per school year group) and 30 adults were included in the analysis |
| 2 | Research question:  Added research question | n/a | We included an exploratory analysis of eye movements in Year 1 that was not preregistered (see Appendix S2). |
| 3 | Study design: Added new control sample of text materials to address reviewer comments. | n/a | We compared the linguistic content of the subtitles in our experiment to three external benchmarks (see Materials and Table S2). |
| 4 | Research question: Added research questions and analyses to address reviewer comments. | n/a | We included an exploratory comparison between Years (Tables S7-S8) and the analysis of fixations within each school year group (Table S3). We also explored whether higher comprehension accuracy was associated with changes in fixations in the subtitle region (Table S12). |
| 5 | Research question: Added analyses to address Editor comments. | n/a | We refitted the models, including random intercepts for video (to ensure findings held across different videos) and for participants’ school (as children were nested within multiple schools). We also repeated the analyses using BPVS instead of TOWRE (Tables S19-S24). |

Table S2. Word length and frequency in the clips from the experiment, as well as in three external benchmarks for 6-to-7-year-old children.

|  |  | Average word length in letters (SD) | Average word frequency, per million  (SD) |
| --- | --- | --- | --- |
| Clips from the experiment | Taking Flight (2016) | 4.0 (1.7) | 6,730 (9,953) |
|  | The Road to El Dorado (2000) | 3.8 (1.8) | 7,536 (10,488) |
|  | Rescuers Down Under (1990) | 4.2 (1.9) | 6,371 (9,608) |
|  | The Wild (2006) | 4.1 (1.9) | 7,055 (10,199) |
| **Words across all clips** | | **4.0 (1.8)** | **6,926 (10,069)** |
| The Little Wandle Reading Fluency Scheme books | Blaise and Flint | 4.2 (1.9) | 6,243 (10,401) |
|  | Cycling in Summer | 3.8 (1.9) | 8,422 (11,705) |
|  | Poetry is Not for Me | 4.0 (1.9) | 6,244 (9,830) |
|  | Talk to the Tail | 4.2 (2.1) | 6,879 (10,589) |
| **Words across all books** | | **4.1 (1.9)** | **6,964 (10,710)** |
| Curriculum-based books | Giraffes Can't Dance | 4.2 (1.9) | 6,459 (10,217) |
|  | Esio Trot | 4.0 (1.9) | 6,028 (9,557) |
|  | The Rainbow Fish | 4.2 (1.9) | 6,993 (11,313) |
| **Words across all books** | | **4.1 (1.9)** | **6,171 (9,826)** |
| CBBC programmes | Danger Mouse | 4.4 (2.3) | 6,765 (10,370) |
|  | The Deep | 4.2 (2.1) | 7,180 (10,266) |
|  | Horrible Histories | 4.2 (2.1) | 6,601 (9,889) |
|  | The Dumping Ground | 4.0 (1.9) | 7,471 (9,975) |
| **Words across all programmes** | | **4.2 (2.1)** | **6,966 (10,076)** |

Note: The Little Wandle Reading Fluency Scheme books are the four ‘Level 1’ books designed for children immediately after the national phonics screen at the end of Year 1. This scheme is used by over 3000 schools across England, representing approximately 20% of all schools. Curriculum-based books are part of the mandatory reading curriculum in the schools from which the children were recruited. The books *Giraffes Can't Dance* and *Esio Trot* are designated for the summer term of Year 1, while *The Rainbow Fish* is introduced in the autumn term of Year 2. CBBC programmes are four of the most popular television programs from the BBC children's television channel CBBC.

Table S3. Summaries of LMMs that estimate the effects of condition (subtitles – 1 or no-subtitles – 0) and school year (coded with treatment contrasts; Year 1 – 0) on the number of fixations and total time reading in the subtitle region in children. Significant effects are shown in bold font.

|  | **Number of fixations**  **(subtitle region; nested contrasts)** | | | | **Total fixation duration**  **(subtitle region; nested contrasts)** | | | | **Probability of a crossover**  **(nested contrasts)** | | | |
| --- | --- | --- | --- | --- | --- | --- | --- | --- | --- | --- | --- | --- |
| *Predictors* | *Est* | *CI* | *t* | *p* | *Est* | *CI* | *t* | *p* | *L-O* | *CI* | *z* | *p* |
| (Intercept) | 0.16 | -0.04 – 0.36 | 1.61 | 0.107 | 46.86 | 1.19 – 92.52 | 2.01 | **0.044** | -3.15 | -3.23 – -3.08 | -85.88 | **<0.001** |
| Year2 | 0.01 | -0.22 – 0.24 | 0.06 | 0.952 | 2.86 | -46.82 – 52.54 | 0.11 | 0.910 | 0.13 | 0.03 – 0.23 | 2.52 | **0.012** |
| Year3 | 0.04 | -0.19 – 0.27 | 0.32 | 0.752 | 8.52 | -41.16 – 58.20 | 0.34 | 0.737 | 0.17 | 0.07 – 0.26 | 3.38 | **0.001** |
| Year4 | 0.00 | -0.23 – 0.23 | 0.03 | 0.974 | -2.47 | -52.15 – 47.21 | -0.10 | 0.922 | 0.04 | -0.06 – 0.14 | 0.86 | 0.390 |
| Year5 | -0.01 | -0.24 – 0.22 | -0.10 | 0.923 | -4.76 | -54.44 – 44.92 | -0.19 | 0.851 | -0.07 | -0.17 – 0.04 | -1.24 | 0.214 |
| Year6 | -0.03 | -0.26 – 0.20 | -0.23 | 0.820 | -9.03 | -58.71 – 40.65 | -0.36 | 0.722 | -0.04 | -0.14 – 0.06 | -0.78 | 0.437 |
| Year1:subtitle | 1.15 | 1.09 – 1.20 | 40.97 | **<0.001** | 280.02 | 266.98 – 293.06 | 42.09 | **<0.001** | 1.36 | 1.28 – 1.45 | 32.92 | **<0.001** |
| Year2:subtitle | 2.15 | 2.10 – 2.21 | 76.73 | **<0.001** | 484.85 | 471.81 – 497.89 | 72.87 | **<0.001** | 1.74 | 1.67 – 1.82 | 45.34 | **<0.001** |
| Year3:subtitle | 2.80 | 2.75 – 2.86 | 99.85 | **<0.001** | 593.39 | 580.35 – 606.43 | 89.18 | **<0.001** | 1.82 | 1.75 – 1.89 | 50.78 | **<0.001** |
| Year4:subtitle | 2.99 | 2.93 – 3.04 | 106.54 | **<0.001** | 626.67 | 613.63 – 639.71 | 94.19 | **<0.001** | 2.09 | 2.02 – 2.16 | 55.92 | **<0.001** |
| Year5:subtitle | 2.94 | 2.89 – 3.00 | 104.83 | **<0.001** | 599.71 | 586.67 – 612.76 | 90.13 | **<0.001** | 2.30 | 2.22 – 2.38 | 57.91 | **<0.001** |
| Year6:subtitle | 2.73 | 2.68 – 2.79 | 97.38 | **<0.001** | 599.47 | 586.43 – 612.51 | 90.10 | **<0.001** | 2.34 | 2.26 – 2.42 | 58.41 | **<0.001** |
| **Random Effects** | | | | | | | | | | | | |
| σ^2^ | 2.13 | | | | 119500.62 | | | |  | | | |
| τ_00_ | 0.55 _item_ | | | | 33801.07 _item_ | | | |  | | | |
|  | 0.19 _participant_ | | | | 8978.46 _participant_ | | | |  | | | |
|  | 0.01 _videofilename_ | | | | 506.13 _videofilename_ | | | |  | | | |
| ICC | 0.26 | | | | 0.27 | | | |  | | | |
| N | 180 _participant_ | | | | 180 _participant_ | | | |  | | | |
|  | 365 _item_ | | | | 365 _item_ | | | |  | | | |
|  | 4 _videofilename_ | | | | 4 _videofilename_ | | | |  | | | |
| Observations | 65700 | | | | 65700 | | | | 271472 | | | |
| Marginal R^2^ / Conditional R^2^ | 0.375 / 0.538 | | | | 0.323 / 0.503 | | | | R^2^ Tjur = 0.085 | | | |

Note: “Item” refers to “subtitle”. Marginal R^2^ indicates variance explained by fixed factors, and conditional R^2^ indicates variance explained by both fixed and random factors. “R^2^ Tjur” is the coefficient of discrimination (equals 1 for a model with perfect discriminating power).

Table S4. Summaries of LMMs that estimate the effects of condition (subtitles or no-subtitles), school year, and TOWRE reading scores on the number of fixations and total time reading in the subtitle and the main scene regions in children. Significant effects are shown in bold font.

|  | **Number of fixations (subtitle region)** | | | | **Total fixation duration**  **(subtitle region)** | | | | **Number of fixations**  **(main scene region)** | | | | **Total fixation duration**  **(main scene region)** | | | |
| --- | --- | --- | --- | --- | --- | --- | --- | --- | --- | --- | --- | --- | --- | --- | --- | --- |
| *Predictors* | *Est* | *CI* | *t* | *p* | *Est* | *CI* | *t* | *p* | *Est* | *CI* | *t* | *p* | *Est* | *CI* | *t* | *p* |
| (Intercept) | 1.41 | 1.19 – 1.62 | 12.96 | **<0.001** | 312.62 | 268.49 – 356.76 | 13.88 | **<0.001** | 4.01 | 3.47 – 4.54 | 14.71 | **<0.001** | 1192.39 | 1028.80 – 1355.98 | 14.29 | **<0.001** |
| condition (subtitles) | 1.23 | 1.22 – 1.24 | 218.23 | **<0.001** | 265.29 | 262.66 – 267.92 | 197.66 | **<0.001** | -0.64 | -0.69 – -0.58 | -22.91 | **<0.001** | -273.03 | -292.68 – -253.37 | -27.23 | **<0.001** |
| school year | 0.01 | -0.04 – 0.05 | 0.22 | 0.824 | -1.03 | -11.27 – 9.21 | -0.20 | 0.844 | 0.01 | -0.05 – 0.08 | 0.39 | 0.695 | -1.70 | -15.36 – 11.96 | -0.24 | 0.807 |
| TOWRE | 0.01 | 0.01 – 0.02 | 9.00 | **<0.001** | 2.59 | 1.96 – 3.21 | 8.07 | **<0.001** | -0.01 | -0.01 – -0.00 | -2.60 | **0.009** | -2.31 | -3.15 – -1.47 | -5.40 | **<0.001** |
| condition x school year | 0.01 | 0.01 – 0.02 | 3.30 | **0.001** | 1.46 | -0.56 – 3.48 | 1.41 | 0.157 | -0.00 | -0.03 – 0.03 | -0.12 | 0.907 | -6.59 | -16.18 – 3.01 | -1.35 | 0.179 |
| condition x TOWRE | 0.01 | 0.01 – 0.01 | 48.08 | **<0.001** | 2.59 | 2.46 – 2.72 | 39.97 | **<0.001** | -0.01 | -0.01 – -0.00 | -6.52 | **<0.001** | -2.86 | -3.46 – -2.26 | -9.32 | **<0.001** |
| **Random Effects** | | | | | | | | | | | | | | | | |
| σ^2^ | 2.06 | | | | 116712.93 | | | | 2.18 | | | | 124926.53 | | | |
| τ_00_ | 0.56 _item_ | | | | 33773.88 _item_ | | | | 3.01 _item_ | | | | 374311.80 _item_ | | | |
|  | 0.16 _participant_ | | | | 7653.09 _participant_ | | | | 0.30 _participant_ | | | | 13764.45 _participant_ | | | |
|  | 0.00 _school_ | | | |  | | | |  | | | | 660.03 _school_ | | | |
|  | 0.04 _videofilename_ | | | | 1467.80 _videofilename_ | | | | 0.26 _videofilename_ | | | | 22505.56 _videofilename_ | | | |
| τ_11_ | 0.01 _item.school_yearc_ | | | | 422.18 _item.school_year_ | | | | 0.13 _item.condition_ | | | | 21880.46 _item.condition_ | | | |
|  |  | | | |  | | | | 0.00 _item.school_year_ | | | | 699.86 _item.school_year_ | | | |
|  |  | | | |  | | | | 0.07 _participant.condition_ | | | | 6961.27 _participant.condition_ | | | |
| ρ_01_ | 0.52 _item_ | | | | 0.33 _item_ | | | | -0.53 _item.condition_ | | | | -0.64 _item.condition_ | | | |
|  |  | | | |  | | | | -0.09 _item.school_year_ | | | | -0.29 _item.school_year_ | | | |
|  |  | | | |  | | | | 0.10 _participant_ | | | | 0.62 _participant_ | | | |
| ICC | 0.28 | | | | 0.27 | | | | 0.63 | | | | 0.78 | | | |
| N | 180 _participant_ | | | | 180 _participant_ | | | | 180 _participant_ | | | | 180 _participant_ | | | |
|  | 365 _item_ | | | | 365 _item_ | | | | 365 _item_ | | | | 365 _item_ | | | |
|  | 4 _videofilename_ | | | | 4 _videofilename_ | | | | 4 _videofilename_ | | | | 4 _videofilename_ | | | |
|  | 5 _school_ | | | |  | | | |  | | | | 5 _school_ | | | |
| Observations | 65700 | | | | 65700 | | | | 65700 | | | | 65700 | | | |
| Marginal R^2^ / Conditional R^2^ | 0.385 / 0.556 | | | | 0.333 / 0.516 | | | | 0.071 / 0.661 | | | | 0.132 / 0.809 | | | |

Note: “Item” refers to “subtitle”. Marginal R^2^ indicates variance explained by fixed factors, and conditional R^2^ indicates variance explained by both fixed and random factors. The analysis of condition-by-year interaction for the number of fixations in the subtitle region showed no effect of year in the subtitle (*β* = 0.02; 95% CI = [-0.03, 0.07]; *t* = 0.80; *p* = .42) or no-subtitle (*β* = -0.01; 95% CI = [-0.06, 0.04]; *t* = -0.37; *p* = .72) conditions. In the subtitle region, the analysis of condition-by-TOWRE interaction showed the effect of TOWRE on the number of fixations (*β* = 0.03; 95% CI = [0.02, 0.03]; *t* = 17.60; *p* < .001) and total fixation duration (*β* = 5.17; 95% CI = [4.53, 5.82]; *t* = 15.83; *p* < .001) for the subtitle condition and no such effects for the no-subtitle condition (*β* = 0.00; 95% CI = [-0.00, 0.00]; *t* = 0.11; *p* = .92 for the number of fixations; *β* = -0.00; 95% CI = [-0.65, 0.64]; *t* = -0.01; *p* = .99 for total fixation duration).

Table S5. Summaries of GLMMs and GLMs that estimate the condition (subtitles or no-subtitles), school year, and TOWRE reading scores on the number of crossovers between the subtitle and the main scene regions in children. Significant effects are shown in bold font.

|  | **Probability of a crossover**  **(interaction)** | | | | **Probability of a crossover**  **(nested contrasts)** | | | |
| --- | --- | --- | --- | --- | --- | --- | --- | --- |
| *Predictors* | *L-O* | *CI* | *z* | *p* | *L-O* | *CI* | *z* | *p* |
| (Intercept) | -2.14 | -2.15 – -2.12 | -270.29 | **<0.001** | -2.14 | -2.15 – -2.12 | -270.29 | **<0.001** |
| condition (subtitles) | 0.98 | 0.96 – 0.99 | 123.44 | **<0.001** | 0.98 | 0.96 – 0.99 | 123.44 | **<0.001** |
| school year | 0.03 | 0.02 – 0.05 | 5.62 | **<0.001** |  |  |  |  |
| TOWRE | 0.00 | 0.00 – 0.00 | 8.88 | **<0.001** |  |  |  |  |
| condition x school year | 0.05 | 0.04 – 0.06 | 8.62 | **<0.001** |  |  |  |  |
| condition x TOWRE | 0.00 | 0.00 – 0.00 | 10.80 | **<0.001** |  |  |  |  |
| subtitles : school year |  |  |  |  | 0.09 | 0.08 – 0.10 | 18.87 | **<0.001** |
| no-subtitles : school year |  |  |  |  | -0.02 | -0.04 – 0.00 | -1.62 | 0.105 |
| subtitles : TOWRE |  |  |  |  | 0.01 | 0.01 – 0.01 | 24.44 | **<0.001** |
| no-subtitles : TOWRE |  |  |  |  | -0.00 | -0.00 – 0.00 | -1.05 | 0.293 |
| Observations | 271472 | | | | 271472 | | | |
| R^2^ Tjur | 0.087 | | | | 0.087 | | | |

Note: “L-O” refers to “log odds”. “R^2^ Tjur” is the coefficient of discrimination (equals 1 for a model with perfect discriminating power).

Table S6. Summaries of GLMMs that estimate the effects of school year and TOWRE reading scores on the probability of skipping subtitles in children. Significant effects are shown in bold font.

|  | **Probability of skipping subtitles** | | | |
| --- | --- | --- | --- | --- |
| *Predictors* | *L-O* | *CI* | *z* | *p* |
| (Intercept) | -1.58 | -2.09 – -1.08 | -6.13 | **<0.001** |
| school year | -0.22 | -0.34 – -0.10 | -3.54 | **<0.001** |
| TOWRE | -0.04 | -0.04 – -0.03 | -9.43 | **<0.001** |
| **Random Effects** | | | | |
| σ^2^ | 3.29 | | | |
| τ_00_ _item_ | 1.61 | | | |
| τ_00_ _participant_ | 1.06 | | | |
| τ_00_ _videofilename_ | 0.22 | | | |
| τ_11_ _item.school_year_ | 0.03 | | | |
| ρ_01_ _item_ | 0.03 | | | |
| ICC | 0.48 | | | |
| N _participant_ | 180 | | | |
| N _item_ | 365 | | | |
| N _videofilename_ | 4 | | | |
| Observations | 32850 | | | |
| Marginal R^2^ / Conditional R^2^ | 0.201 / 0.582 | | | |

Note: “Item” refers to “subtitle”. “L-O” refers to “log odds”. Marginal R^2^ indicates variance explained by fixed factors, and conditional R^2^ indicates variance explained by both fixed and random factors.

Table S7. Summaries of LMMs that estimate the effects of condition (subtitles or no-subtitles) and school year (coded with successive differences contrasts) on the number of fixations and total time reading in the subtitle region in children. Significant effects are shown in bold font.

|  | **Number of fixations (subtitle region; interaction)** | | | | **Number of fixations (subtitle region; nested contrasts)** | | | | **Total fixation duration**  **(subtitle region; interaction)** | | | | **Total fixation duration**  **(subtitle region; nested contrasts)** | | | |
| --- | --- | --- | --- | --- | --- | --- | --- | --- | --- | --- | --- | --- | --- | --- | --- | --- |
| *Predictors* | *Est* | *CI* | *t* | *p* | *Est* | *CI* | *t* | *p* | *Est* | *CI* | *t* | *p* | *Est* | *CI* | *t* | *p* |
| (Intercept) | 1.40 | 1.26 – 1.53 | 20.47 | **<0.001** | 1.40 | 1.26 – 1.53 | 20.47 | **<0.001** | 311.39 | 278.98 – 343.79 | 18.83 | **<0.001** | 311.39 | 278.98 – 343.79 | 18.83 | **<0.001** |
| condition (subtitles) | 1.23 | 1.22 – 1.24 | 214.85 | **<0.001** | 1.23 | 1.22 – 1.24 | 214.85 | **<0.001** | 265.34 | 262.68 – 268.00 | 195.37 | **<0.001** | 265.34 | 262.68 – 268.00 | 195.37 | **<0.001** |
| Years 2-1 | 0.51 | 0.28 – 0.73 | 4.42 | **<0.001** |  |  |  |  | 105.28 | 56.46 – 154.10 | 4.23 | **<0.001** |  |  |  |  |
| Years 3-2 | 0.35 | 0.13 – 0.58 | 3.08 | **0.002** |  |  |  |  | 59.92 | 11.11 – 108.74 | 2.41 | **0.016** |  |  |  |  |
| Years 4-3 | 0.06 | -0.16 – 0.29 | 0.53 | 0.598 |  |  |  |  | 5.65 | -43.17 – 54.47 | 0.23 | 0.820 |  |  |  |  |
| Years 5-4 | -0.04 | -0.26 – 0.19 | -0.34 | 0.735 |  |  |  |  | -15.76 | -64.58 – 33.05 | -0.63 | 0.527 |  |  |  |  |
| Years 6-5 | -0.12 | -0.35 – 0.11 | -1.04 | 0.297 |  |  |  |  | -4.39 | -53.21 – 44.42 | -0.18 | 0.860 |  |  |  |  |
| condition x Years 2-1 | 0.50 | 0.46 – 0.54 | 25.28 | **<0.001** |  |  |  |  | 102.42 | 93.19 – 111.64 | 21.77 | **<0.001** |  |  |  |  |
| condition x Years 3-2 | 0.32 | 0.29 – 0.36 | 16.35 | **<0.001** |  |  |  |  | 54.27 | 45.05 – 63.49 | 11.54 | **<0.001** |  |  |  |  |
| condition x Years 4-3 | 0.09 | 0.05 – 0.13 | 4.73 | **<0.001** |  |  |  |  | 16.64 | 7.42 – 25.86 | 3.54 | **<0.001** |  |  |  |  |
| condition x Years 5-4 | -0.02 | -0.06 – 0.01 | -1.21 | 0.227 |  |  |  |  | -13.48 | -22.70 – -4.26 | -2.86 | **0.004** |  |  |  |  |
| condition x Years 6-5 | -0.10 | -0.14 – -0.07 | -5.27 | **<0.001** |  |  |  |  | -0.12 | -9.34 – 9.10 | -0.03 | 0.979 |  |  |  |  |
| subtitle:Years 2-1 |  |  |  |  | 1.01 | 0.78 – 1.24 | 8.65 | **<0.001** |  |  |  |  | 207.69 | 158.01 – 257.37 | 8.19 | **<0.001** |
| nosubtitle:Years 2-1 |  |  |  |  | 0.01 | -0.22 – 0.24 | 0.06 | 0.952 |  |  |  |  | 2.86 | -46.82 – 52.54 | 0.11 | 0.910 |
| subtitle:Years 3-2 |  |  |  |  | 0.68 | 0.45 – 0.91 | 5.81 | **<0.001** |  |  |  |  | 114.20 | 64.51 – 163.88 | 4.51 | **<0.001** |
| nosubtitle:Years 3-2 |  |  |  |  | 0.03 | -0.20 – 0.26 | 0.26 | 0.798 |  |  |  |  | 5.65 | -44.03 – 55.33 | 0.22 | 0.824 |
| subtitle:Years 4-3 |  |  |  |  | 0.15 | -0.07 – 0.38 | 1.32 | 0.186 |  |  |  |  | 22.29 | -27.39 – 71.97 | 0.88 | 0.379 |
| nosubtitle:Years 4-3 |  |  |  |  | -0.03 | -0.26 – 0.20 | -0.28 | 0.776 |  |  |  |  | -10.99 | -60.67 – 38.69 | -0.43 | 0.665 |
| subtitle:Years 5-4 |  |  |  |  | -0.06 | -0.29 – 0.17 | -0.54 | 0.590 |  |  |  |  | -29.24 | -78.92 – 20.44 | -1.15 | 0.249 |
| nosubtitle:Years 5-4 |  |  |  |  | -0.02 | -0.24 – 0.21 | -0.13 | 0.898 |  |  |  |  | -2.29 | -51.97 – 47.39 | -0.09 | 0.928 |
| subtitle:Years 6-5 |  |  |  |  | -0.22 | -0.45 – 0.00 | -1.92 | 0.055 |  |  |  |  | -4.51 | -54.20 – 45.17 | -0.18 | 0.859 |
| nosubtitle:Years 6-5 |  |  |  |  | -0.02 | -0.24 – 0.21 | -0.13 | 0.895 |  |  |  |  | -4.27 | -53.95 – 45.41 | -0.17 | 0.866 |
| **Random Effects** | | | | | | | | | | | | | | | | |
| σ^2^ | 2.13 | | | | 2.13 | | | | 119500.62 | | | | 119500.62 | | | |
| τ_00_ | 0.55 _item_ | | | | 0.55 _item_ | | | | 33801.03 _item_ | | | | 33801.07 _item_ | | | |
|  | 0.19 _participant_ | | | | 0.19 _participant_ | | | | 8978.45 _participant_ | | | | 8978.46 _participant_ | | | |
|  | 0.01 _videofilename_ | | | | 0.01 _videofilename_ | | | | 506.14 _videofilename_ | | | | 506.14 _videofilename_ | | | |
| ICC | 0.26 | | | | 0.26 | | | | 0.27 | | | | 0.27 | | | |
| N | 180 _participant_ | | | | 180 _participant_ | | | | 180 _participant_ | | | | 180 _participant_ | | | |
|  | 365 _item_ | | | | 365 _item_ | | | | 365 _item_ | | | | 365 _item_ | | | |
|  | 4 _videofilename_ | | | | 4 _videofilename_ | | | | 4 _videofilename_ | | | | 4 _videofilename_ | | | |
| Observations | 65700 | | | | 65700 | | | | 65700 | | | | 65700 | | | |
| Marginal R^2^ / Conditional R^2^ | 0.375 / 0.538 | | | | 0.375 / 0.538 | | | | 0.323 / 0.503 | | | | 0.323 / 0.503 | | | |

Note: “Item” refers to “subtitle”. Marginal R^2^ indicates variance explained by fixed factors, and conditional R^2^ indicates variance explained by both fixed and random factors.

Table S8. Summaries of GLMMs and GLMs that estimate the condition (subtitles or no-subtitles) and school year (coded with successive differences contrasts) on the number of crossovers between the subtitle and the main scene regions and the probability of skipping subtitles in children. Significant effects are shown in bold font.

|  | **Probability of a crossover**  **(interaction)** | | | | **Probability of a crossover**  **(nested contrasts)** | | | | **Probability of skipping subtitles** | | | |
| --- | --- | --- | --- | --- | --- | --- | --- | --- | --- | --- | --- | --- |
| *Predictors* | *L-O* | *CI* | *z* | *p* | *L-O* | *CI* | *z* | *p* | *L-O* | *CI* | *z* | *p* |
| (Intercept) | -2.14 | -2.16 – -2.13 | -270.31 | **<0.001** | -2.14 | -2.16 – -2.13 | -270.31 | **<0.001** | -1.57 | -2.09 – -1.06 | -5.97 | **<0.001** |
| condition (subtitles) | 0.97 | 0.96 – 0.99 | 122.48 | **<0.001** | 0.97 | 0.96 – 0.99 | 122.48 | **<0.001** |  |  |  |  |
| Years 2-1 | 0.32 | 0.26 – 0.37 | 11.19 | **<0.001** |  |  |  |  | -1.58 | -2.18 – -0.98 | -5.14 | **<0.001** |
| Years 3-2 | 0.08 | 0.03 – 0.13 | 3.07 | **0.002** |  |  |  |  | -0.88 | -1.48 – -0.27 | -2.85 | **0.004** |
| Years 4-3 | 0.01 | -0.04 – 0.06 | 0.36 | 0.722 |  |  |  |  | -0.45 | -1.06 – 0.15 | -1.46 | 0.144 |
| Years 5-4 | -0.00 | -0.06 – 0.05 | -0.11 | 0.914 |  |  |  |  | -0.10 | -0.71 – 0.51 | -0.32 | 0.747 |
| Years 6-5 | 0.04 | -0.01 – 0.10 | 1.55 | 0.120 |  |  |  |  | -0.10 | -0.70 – 0.51 | -0.31 | 0.755 |
| condition x Years 2-1 | 0.19 | 0.13 – 0.24 | 6.65 | **<0.001** |  |  |  |  |  |  |  |  |
| condition x Years 3-2 | 0.04 | -0.01 – 0.09 | 1.59 | 0.111 |  |  |  |  |  |  |  |  |
| condition x Years 4-3 | 0.13 | 0.08 – 0.18 | 5.12 | **<0.001** |  |  |  |  |  |  |  |  |
| condition x Years 5-4 | 0.11 | 0.05 – 0.16 | 3.88 | **<0.001** |  |  |  |  |  |  |  |  |
| condition x Years 6-5 | 0.02 | -0.04 – 0.07 | 0.70 | 0.486 |  |  |  |  |  |  |  |  |
| subtitle:Years 2-1 |  |  |  |  | 0.50 | 0.46 – 0.55 | 20.35 | **<0.001** |  |  |  |  |
| nosubtitle:Years 2-1 |  |  |  |  | 0.13 | 0.03 – 0.23 | 2.52 | **0.012** |  |  |  |  |
| subtitle:Years 3-2 |  |  |  |  | 0.12 | 0.08 – 0.16 | 5.81 | **<0.001** |  |  |  |  |
| nosubtitle:Years 3-2 |  |  |  |  | 0.04 | -0.06 – 0.13 | 0.81 | 0.419 |  |  |  |  |
| subtitle:Years 4-3 |  |  |  |  | 0.14 | 0.10 – 0.18 | 7.26 | **<0.001** |  |  |  |  |
| nosubtitle:Years 4-3 |  |  |  |  | -0.12 | -0.22 – -0.03 | -2.57 | **0.010** |  |  |  |  |
| subtitle:Years 5-4 |  |  |  |  | 0.10 | 0.07 – 0.14 | 5.42 | **<0.001** |  |  |  |  |
| nosubtitle:Years 5-4 |  |  |  |  | -0.11 | -0.21 – -0.01 | -2.13 | **0.034** |  |  |  |  |
| subtitle:Years 6-5 |  |  |  |  | 0.06 | 0.03 – 0.10 | 3.35 | **0.001** |  |  |  |  |
| nosubtitle:Years 6-5 |  |  |  |  | 0.02 | -0.08 – 0.13 | 0.46 | 0.649 |  |  |  |  |
| **Random Effects** | | | | | | | | | | | | |
| σ^2^ |  | | | |  | | | | 3.29 | | | |
| τ_00_ |  | | | |  | | | | 1.60 _item_ | | | |
|  |  | | | |  | | | | 1.37 _participant_ | | | |
|  |  | | | |  | | | | 0.23 _videofilename_ | | | |
| ICC |  | | | |  | | | | 0.49 | | | |
| N |  | | | |  | | | | 180 _participant_ | | | |
|  |  | | | |  | | | | 365 _item_ | | | |
|  |  | | | |  | | | | 4 _videofilename_ | | | |
| Observations | 271472 | | | | 271472 | | | | 32850 | | | |
| R^2^ Tjur | 0.085 | | | | 0.085 | | | | 0.157 / 0.572 | | | |

Note: “Item” refers to “subtitle”. “L-O” refers to “log odds”. “R^2^ Tjur” is the coefficient of discrimination (equals 1 for a model with perfect discriminating power).

Table S9. Summaries of LMMs that estimate the effects of word length and frequency and their interaction with school year and TOWRE reading scores on fixation durations in children. Significant effects are shown in bold font.

|  | **Gaze duration** | | | | **Total fixation duration** | | | |
| --- | --- | --- | --- | --- | --- | --- | --- | --- |
| *Predictors* | *Est.* | *CI* | *t* | *p* | *Est.* | *CI* | *t* | *p* |
| (Intercept) | 5.53 | 5.51 – 5.56 | 442.08 | **<0.001** | 5.64 | 5.62 – 5.67 | 435.35 | **<0.001** |
| length | 0.03 | 0.03 – 0.04 | 10.22 | **<0.001** | 0.04 | 0.04 – 0.05 | 10.68 | **<0.001** |
| frequency | -0.02 | -0.02 – -0.01 | -8.94 | **<0.001** | -0.02 | -0.02 – -0.01 | -8.82 | **<0.001** |
| school year | -0.04 | -0.05 – -0.02 | -4.49 | **<0.001** | -0.04 | -0.05 – -0.02 | -4.60 | **<0.001** |
| TOWRE | -0.00 | -0.00 – -0.00 | -5.09 | **<0.001** | -0.00 | -0.00 – -0.00 | -3.86 | **<0.001** |
| school year x length | -0.00 | -0.00 – 0.00 | -0.80 | 0.424 | -0.00 | -0.00 – 0.00 | -0.10 | 0.918 |
| school year x frequency | 0.00 | 0.00 – 0.00 | 2.49 | **0.014** | 0.00 | 0.00 – 0.00 | 3.03 | **0.002** |
| TOWRE x length | -0.00 | -0.00 – 0.00 | -0.60 | 0.546 | 0.00 | -0.00 – 0.00 | 1.12 | 0.262 |
| TOWRE x frequency | -0.00 | -0.00 – 0.00 | -0.43 | 0.665 | -0.00 | -0.00 – 0.00 | -0.45 | 0.655 |
| **Random Effects** | | | | | | | | |
| σ^2^ | 0.24 | | | | 0.29 | | | |
| τ_00_ | 0.02 _item_ | | | | 0.03 _item_ | | | |
|  | 0.02 _participant_ | | | | 0.02 _participant_ | | | |
| τ_11_ | 0.00 _item.school_year_ | | | | 0.00 _item.school_yearc_ | | | |
|  | 0.00 _participant.length_ | | | | 0.00 _participant.length_ | | | |
|  | 0.00 _participant.logfreq_ | | | | 0.00 _participant.logfreq_ | | | |
| ρ_01_ | -0.01 _item_ | | | | 0.05 _item_ | | | |
|  | 0.21 _participant.length_ | | | | 0.20 _participant.length_ | | | |
|  | -0.26 _participant.logfreq_ | | | | -0.21 _participant.logfreq_ | | | |
| ICC | 0.15 | | | | 0.16 | | | |
| N | 180 _participant_ | | | | 180 _participant_ | | | |
|  | 873 _item_ | | | | 873 _item_ | | | |
| Observations | 53920 | | | | 53920 | | | |
| Marginal R^2^ / Conditional R^2^ | 0.073 / 0.212 | | | | 0.075 / 0.225 | | | |

Note: “Item” refers to “word”. Marginal R^2^ indicates variance explained by fixed factors, and conditional R^2^ indicates variance explained by both fixed and random factors.

Table S10. Summaries of LMMs and a GLM that estimate the effects of school year and TOWRE reading scores on the number of fixations, mean fixation duration, and skipping probabilities of words in children. Significant effects are shown in bold font.

|  | **Number of fixations** | | | | **Mean fixation duration** | | | | **Probability of skipping** | | | | **Probability of skipping**  **(word relative position)** | | | |
| --- | --- | --- | --- | --- | --- | --- | --- | --- | --- | --- | --- | --- | --- | --- | --- | --- |
| *Predictors* | *Est.* | *CI* | *t* | *p* | *Est.* | *CI* | *t* | *p* | *L-O* | *CI* | *z* | *p* | *L-O* | *CI* | *z* | *p* |
| (Intercept) | 1.41 | 1.38 – 1.44 | 97.39 | **<0.001** | 5.41 | 5.39 – 5.44 | 431.73 | **<0.001** | 0.16 | 0.15 – 0.17 | 27.73 | **<0.001** | 0.17 | 0.15 – 0.18 | 27.97 | **<0.001** |
| school year | -0.03 | -0.05 – -0.02 | -3.91 | **<0.001** | -0.02 | -0.04 – -0.00 | -2.30 | **0.023** | 0.00 | -0.01 – 0.01 | 0.70 | 0.484 | 0.00 | -0.01 – 0.01 | 0.53 | 0.596 |
| TOWRE | 0.00 | -0.00 – 0.00 | 0.30 | 0.764 | -0.00 | -0.00 – -0.00 | -4.48 | **<0.001** | -0.01 | -0.01 – -0.01 | -28.57 | **<0.001** | -0.01 | -0.01 – -0.01 | -29.41 | **<0.001** |
| word relative position in a sentence |  |  |  |  |  |  |  |  |  |  |  |  | -0.17 | -0.17 – -0.16 | -60.42 | **<0.001** |
| word relative position x TOWRE |  |  |  |  |  |  |  |  |  |  |  |  | 0.00 | 0.00 – 0.00 | 3.32 | **0.001** |
| word relative position x school year |  |  |  |  |  |  |  |  |  |  |  |  | 0.01 | 0.00 – 0.01 | 2.92 | **0.003** |
| **Random Effects** | | | | | | | | | | | | | | | | |
| σ^2^ | 0.44 | | | | 0.18 | | | |  | | | |  | | | |
| τ_00_ | 0.08 _item_ | | | | 0.01 _item_ | | | |  | | | |  | | | |
|  | 0.02 _participant_ | | | | 0.02 _participant_ | | | |  | | | |  | | | |
|  |  | | | | 0.00 _school_ | | | |  | | | |  | | | |
| τ_11_ | 0.00 _item.school_year_ | | | | 0.00 _item.school_yearc_ | | | |  | | | |  | | | |
| ρ_01_ | -0.44 _item_ | | | | -0.03 _item_ | | | |  | | | |  | | | |
| ICC | 0.18 | | | | 0.17 | | | |  | | | |  | | | |
| N | 180 _participant_ | | | | 180 _participant_ | | | |  | | | |  | | | |
|  | 873 _item_ | | | | 873 _item_ | | | |  | | | |  | | | |
|  |  | | | | 5 _school_ | | | |  | | | |  | | | |
| Observations | 53920 | | | | 53920 | | | | 118716 | | | | 118716 | | | |
| Marginal R^2^ / Conditional R^2^ | 0.004 / 0.184 | | | | 0.022 / 0.184 | | | | 0.010 | | | | 0.042 | | | |

Note: “Item” refers to “word”. “L-O” refers to “log odds”. Marginal R^2^ indicates variance explained by fixed factors, and conditional R^2^ indicates variance explained by both fixed and random factors.

Table S11. Summaries of LMMs that estimate the effects of condition (subtitles or no-subtitles), school year, and TOWRE reading scores on response accuracy to comprehension questions in children. Significant effects are shown in bold font.

|  | **Accuracy** | | | |
| --- | --- | --- | --- | --- |
| *Predictors* | *Est.* | *CI* | *t* | *p* |
| (Intercept) | 15.33 | 14.35 – 16.32 | 30.53 | **<0.001** |
| condition (subtitles) | 0.08 | -0.08 – 0.25 | 0.97 | 0.334 |
| school year | 0.67 | 0.35 – 0.99 | 4.11 | **<0.001** |
| TOWRE | 0.04 | 0.02 – 0.05 | 4.75 | **<0.001** |
| condition x school year | -0.08 | -0.20 – 0.05 | -1.16 | 0.245 |
| condition x TOWRE | 0.01 | 0.00 – 0.02 | 2.16 | **0.032** |
| **Random Effects** | | | | |
| σ^2^ | 4.33 | | | |
| τ_00_ _participant_ | 3.28 | | | |
| τ_00_ _school_ | 0.10 | | | |
| τ_00_ _videofilename_ | 0.77 | | | |
| τ_11_ _participant.condition_ | 0.21 | | | |
| τ_11_ _videofilename.school_year_ | 0.05 | | | |
| ρ_01_ _participant_ | -0.12 | | | |
| ρ_01_ _videofilename_ | -0.98 | | | |
| ICC | 0.51 | | | |
| N _participant_ | 180 | | | |
| N _videofilename_ | 4 | | | |
| N _school_ | 5 | | | |
| Observations | 720 | | | |
| Marginal R^2^ / Conditional R^2^ | 0.299 / 0.657 | | | |

Note: Marginal R^2^ indicates variance explained by fixed factors, and conditional R^2^ indicates variance explained by both fixed and random factors.

Table S12. Spearman correlation for the children’s response accuracy to comprehension questions and the total number of fixations or total fixation duration in the subtitle region in the subtitle condition. Significant effects are shown in bold font.

|  | **comprehension ~ number of fixations** | **comprehension ~ total fixation duration** |
| --- | --- | --- |
| Year 1 | *r* = 0.18, *p* = 0.15 | *r* = 0.12, *p* = 0.36 |
| Year 2 | *r* = 0.14 , *p* = 0.29 | *r* = 0.07, *p* = 0.58 |
| Year 3 | *r* = 0.20, *p* =0.13 | *r* = 0.18, *p* = 0.16 |
| Year 4 | *r* = 0.21, *p* = 0.10 | *r* = 0.21, *p* = 0.11 |
| Year 5 | ***r* = 0.38, *p* = .003** | ***r* = 0.42, *p* < .001** |
| Year 6 | *r* = 0.16, *p* = 0.24 | *r* = 0.03, *p* = 0.84 |
| All groups | ***r* = 0.40, *p* < .001** | ***r* = 0.35, *p* < .001** |

Table S13. Summaries of LMMs that estimate the effects of condition (subtitles or no-subtitles) and group (children in Year 6 or adults) on the number of fixations and total time reading in the subtitle and the main scene regions. Significant effects are shown in bold font.

|  | **Number of fixations**  **(subtitle region)** | | | | **Total fixation duration**  **(subtitle region)** | | | | **Number of fixations**  **(main scene region)** | | | | **Total fixation duration**  **(main scene region)** | | | |
| --- | --- | --- | --- | --- | --- | --- | --- | --- | --- | --- | --- | --- | --- | --- | --- | --- |
| *Predictors* | *Est* | *CI* | *t* | *p* | *Est* | *CI* | *t* | *p* | *Est* | *CI* | *t* | *p* | *Est* | *CI* | *t* | *p* |
| (Intercept) | 1.43 | 1.30 – 1.57 | 21.11 | **<0.001** | 304.80 | 273.92 – 335.68 | 19.34 | **<0.001** | 4.07 | 3.60 – 4.53 | 16.99 | **<0.001** | 1188.46 | 1067.23 – 1309.68 | 19.21 | **<0.001** |
| condition (subtitles) | 1.31 | 1.24 – 1.37 | 37.64 | **<0.001** | 270.52 | 255.64 – 285.40 | 35.63 | **<0.001** | -0.64 | -0.72 – -0.57 | -16.96 | **<0.001** | -297.11 | -322.82 – -271.40 | -22.65 | **<0.001** |
| adults | -0.07 | -0.16 – 0.01 | -1.65 | 0.100 | -33.23 | -53.21 – -13.25 | -3.26 | **0.001** | 0.11 | -0.04 – 0.27 | 1.41 | 0.158 | 16.38 | -9.43 – 42.19 | 1.24 | 0.214 |
| condition x adults | -0.06 | -0.07 – -0.04 | -7.56 | **<0.001** | -29.70 | -33.42 – -25.98 | -15.64 | **<0.001** | 0.05 | -0.01 – 0.11 | 1.54 | 0.125 | 17.31 | -2.94 – 37.56 | 1.67 | 0.094 |
| **Random Effects** | | | | | | | | | | | | | | | | |
| σ^2^ | 1.34 | | | | 77821.91 | | | | 2.43 | | | | 103249.91 | | | |
| τ_00_ | 0.53 _item_ | | | | 27924.76 _item_ | | | | 3.10 _item_ | | | | 370374.77 _item_ | | | |
|  | 0.11 _participant_ | | | | 5905.96 _participant_ | | | | 0.36 _participant_ | | | | 9948.45 _participant_ | | | |
|  | 0.01 _videofilename_ | | | | 276.03 _videofilename_ | | | | 0.17 _videofilename_ | | | | 10467.91 _videofilename_ | | | |
| τ_11_ | 0.42 _item.condition_ | | | | 19714.62 _item.condition_ | | | | 0.15 _item.condition_ | | | | 23841.74 _item.condition_ | | | |
|  | 0.01 _item.group_ | | | | 694.13 _item.group_ | | | | 0.02 _item.group_ | | | | 1024.37 _item.group_ | | | |
|  |  | | | |  | | | | 0.06 _participant.condition_ | | | | 6118.72 _participant.condition_ | | | |
| ρ_01_ | 0.96 _item.condition_ | | | | 0.92 _item.condition_ | | | | -0.55 _item.condition_ | | | | -0.66 _item.condition_ | | | |
|  | -0.45 _item.group_ | | | | -0.77 _item.group_ | | | | 0.02 _item.group_ | | | | 0.14 _item.group_ | | | |
|  |  | | | |  | | | | -0.25 _participant_ | | | | 0.22 _participant_ | | | |
| ICC | 0.44 | | | | 0.41 | | | | 0.61 | | | | 0.80 | | | |
| N | 60 _participant_ | | | | 60 _participant_ | | | | 60 _participant_ | | | | 60 _participant_ | | | |
|  | 365 _item_ | | | | 365 _item_ | | | | 365 _item_ | | | | 365 _item_ | | | |
|  | 4 _videofilename_ | | | | 4 _videofilename_ | | | | 4 _videofilename_ | | | | 4 _videofilename_ | | | |
| Observations | 21900 | | | | 21900 | | | | 21900 | | | | 21900 | | | |
| Marginal R^2^ / Conditional R^2^ | 0.417 / 0.676 | | | | 0.362 / 0.625 | | | | 0.064 / 0.639 | | | | 0.145 / 0.832 | | | |

Note: “Item” refers to “subtitle”. Marginal R^2^ indicates variance explained by fixed factors, and conditional R^2^ indicates variance explained by both fixed and random factors.

|  | | | | |
| --- | --- | --- | --- | --- |
|  |  |  |  |  |
|  |  |  |  |  |
|  |  |  |  |  |
|  |  |  |  |  |
|  |  |  |  |  |
|  |  |  |  |  |
|  |  |  |  |  |
|  |  |  |  |  |
|  |  |  |  |  |
|  |  |  |  |  |
|  |  |  |  |  |
|  |  |  |  |  |
|  |  |  |  |  |
|  |  |  |  |  |

Table S14. The summary of GLMM that estimates the effects of condition (subtitles or no-subtitles) and group (children in Year 6 or adults) on the number of crossovers between the subtitle and the main scene regions. Significant effects are shown in bold font.

|  | **Probability of a crossover** | | | |
| --- | --- | --- | --- | --- |
| *Predictors* | *L-O* | *CI* | *z* | *p* |
| (Intercept) | -2.85 | -3.03 – -2.67 | -30.94 | **<0.001** |
| condition (subtitles) | 2.04 | 1.90 – 2.18 | 28.26 | **<0.001** |
| adults | -0.18 | -0.28 – -0.08 | -3.52 | **<0.001** |
| condition x adults | 0.16 | 0.08 – 0.24 | 3.91 | **<0.001** |
| **Random Effects** | | | | |
| σ^2^ | 3.29 | | | |
| τ_00_ _item_ | 0.74 | | | |
| τ_00_ _participant_ | 0.14 | | | |
| τ_00_ _videofilename_ | 0.01 | | | |
| τ_11_ _item.condition_ | 0.82 | | | |
| τ_11_ _participant.condition_ | 0.08 | | | |
| ρ_01_ _item_ | -0.90 | | | |
| ρ_01_ _participant_ | -0.78 | | | |
| ICC | 0.32 | | | |
| N _participant_ | 60 | | | |
| N _item_ | 365 | | | |
| N _videofilename_ | 4 | | | |
| Observations | 89511 | | | |
| Marginal R^2^ / Conditional R^2^ | 0.459 / 0.631 | | | |

Note: “Item” refers to “subtitle”. “L-O” refers to “log odds”. Marginal R^2^ indicates variance explained by fixed factors, and conditional R^2^ indicates variance explained by both fixed and random factors.

Table S15. The summary of GLMM that estimates the effect of group (children in Year 6 or adults) on the probability of skipping subtitles. Significant effects are shown in bold font.

|  | **Probability of skipping subtitles** | | | |
| --- | --- | --- | --- | --- |
| *Predictors* | *L-O* | *CI* | *z* | *p* |
| (Intercept) | -2.32 | -2.65 – -1.99 | -13.61 | **<0.001** |
| adults | 0.08 | -0.19 – 0.34 | 0.59 | 0.558 |
| **Random Effects** | | | | |
| σ^2^ | 3.29 | | | |
| τ_00_ _item_ | 1.33 | | | |
| τ_00_ _participant_ | 1.00 | | | |
| τ_00_ _videofilename_ | 0.03 | | | |
| τ_11_ _item.group1_ | 0.06 | | | |
| ρ_01_ _item_ | -0.12 | | | |
| ICC | 0.42 | | | |
| N _participant_ | 60 | | | |
| N _item_ | 365 | | | |
| N _videofilename_ | 4 | | | |
| Observations | 10950 | | | |
| Marginal R^2^ / Conditional R^2^ | 0.001 / 0.424 | | | |

Note: “Item” refers to “subtitle”. “L-O” refers to “log odds”. Marginal R^2^ indicates variance explained by fixed factors, and conditional R^2^ indicates variance explained by both fixed and random factors.

Table S16. Summaries of LMMs that estimate the effects of length and frequency on fixation durations in adults compared to children in Year 6. Significant effects are shown in bold font.

|  | **Gaze duration** | | | | **Total fixation duration** | | | |
| --- | --- | --- | --- | --- | --- | --- | --- | --- |
| *Predictors* | *Est.* | *CI* | *t* | *p* | *Est.* | *CI* | *t* | *p* |
| (Intercept) | 5.35 | 5.31 – 5.40 | 236.68 | **<0.001** | 5.47 | 5.42 – 5.51 | 235.72 | **<0.001** |
| adults | -0.13 | -0.18 – -0.09 | -6.00 | **<0.001** | -0.14 | -0.18 – -0.09 | -6.20 | **<0.001** |
| length | 0.03 | 0.02 – 0.04 | 7.35 | **<0.001** | 0.04 | 0.03 – 0.05 | 8.52 | **<0.001** |
| frequency | -0.01 | -0.01 – -0.01 | -6.68 | **<0.001** | -0.01 | -0.02 – -0.01 | -6.40 | **<0.001** |
| adults x length | -0.00 | -0.01 – 0.00 | -1.47 | 0.141 | -0.00 | -0.01 – 0.00 | -1.17 | 0.243 |
| adults x frequency | 0.00 | -0.00 – 0.00 | 0.25 | 0.805 | 0.00 | -0.00 – 0.00 | 0.95 | 0.340 |
| **Random Effects** | | | | | | | | |
| σ^2^ | 0.30 | | | | 0.34 | | | |
| τ_00_ | 0.01 _item_ | | | | 0.03 _item_ | | | |
|  | 0.03 _participant_ | | | | 0.03 _participant_ | | | |
| τ_11_ | 0.00 _item.group_ | | | | 0.00 _item.group_ | | | |
|  | 0.00 _participant.length_ | | | | 0.00 _participant.length_ | | | |
| ρ_01_ | 0.10 _item_ | | | | 0.05 _item_ | | | |
|  | 0.61 _participant_ | | | | 0.46 _participant_ | | | |
| ICC | 0.12 | | | | 0.14 | | | |
| N | 60 _participant_ | | | | 60 _participant_ | | | |
|  | 870 _item_ | | | | 870 _item_ | | | |
| Observations | 21015 | | | | 21015 | | | |
| Marginal R^2^ / Conditional R^2^ | 0.069 / 0.182 | | | | 0.079 / 0.211 | | | |

Note: “Item” refers to “word”. Marginal R^2^ indicates variance explained by fixed factors, and conditional R^2^ indicates variance explained by both fixed and random factors.

Table S17. Summaries of LMMs and a GLMM that estimate the effects of group (children in Year 6 or adults) on the number of fixations, mean fixation duration, and skipping probabilities of words. Significant effects are shown in bold font.

|  | **Number of fixations** | | | | **Mean fixation duration** | | | | **Probability of skipping** | | | |
| --- | --- | --- | --- | --- | --- | --- | --- | --- | --- | --- | --- | --- |
| *Predictors* | *Est.* | *CI* | *t* | *p* | *Est.* | *CI* | *t* | *p* | *L-O* | *CI* | *z* | *p* |
| (Intercept) | 1.33 | 1.30 – 1.37 | 81.68 | **<0.001** | 5.28 | 5.23 – 5.32 | 240.82 | **<0.001** | -0.13 | -0.25 – -0.01 | -2.20 | **0.028** |
| adults | -0.03 | -0.06 – -0.00 | -2.06 | **0.039** | -0.12 | -0.16 – -0.08 | -5.62 | **<0.001** | -0.01 | -0.10 – 0.07 | -0.30 | 0.762 |
| **Random Effects** | | | | | | | | | | | | |
| σ^2^ | 0.36 | | | | 0.25 | | | | 3.29 | | | |
| τ_00_ | 0.05 _item_ | | | | 0.01 _item_ | | | | 1.53 _item_ | | | |
|  | 0.01 _participant_ | | | | 0.03 _participant_ | | | | 0.10 _participant_ | | | |
| τ_11_ | 0.00 _item.group_ | | | | 0.00 _item.group_ | | | | 0.05 _item.group_ | | | |
| ρ_01_ | -0.34 _item_ | | | | 0.21 _item_ | | | | -0.18 _item_ | | | |
| ICC | 0.15 | | | | 0.13 | | | | 0.34 | | | |
| N | 60 _participant_ | | | | 60 _participant_ | | | | 60 _participant_ | | | |
|  | 870 _item_ | | | | 870 _item_ | | | | 877 _item_ | | | |
| Observations | 21015 | | | | 21015 | | | | 45955 | | | |
| Marginal R^2^ / Conditional R^2^ | 0.002 / 0.153 | | | | 0.050 / 0.176 | | | | 0.000 / 0.338 | | | |

Note: “Item” refers to “word”. “L-O” refers to “log odds”. Marginal R^2^ indicates variance explained by fixed factors, and conditional R^2^ indicates variance explained by both fixed and random factors.

Table S18. Summary of LMM that estimates the effects of condition (with or without subtitles) and group (children in Year 6 or adults) on response accuracy to comprehension questions. Significant effects are shown in bold font.

|  | **Accuracy** | | | |
| --- | --- | --- | --- | --- |
| *Predictors* | *Est.* | *CI* | *t* | *p* |
| (Intercept) | 17.89 | 17.35 – 18.42 | 65.81 | **<0.001** |
| condition (subtitles) | 0.01 | -0.18 – 0.20 | 0.13 | 0.898 |
| adults | 0.70 | 0.39 – 1.02 | 4.39 | **<0.001** |
| condition x adults | -0.02 | -0.21 – 0.17 | -0.21 | 0.831 |
| **Random Effects** | | | | |
| σ^2^ | 2.29 | | | |
| τ_00_ _participant_ | 0.80 | | | |
| τ_00_ _videofilename_ | 0.20 | | | |
| τ_11_ _videofilename.group_ | 0.01 | | | |
| ρ_01_ _videofilename_ | -0.27 | | | |
| ICC | 0.31 | | | |
| N _participant_ | 60 | | | |
| N _videofilename_ | 4 | | | |
| Observations | 240 | | | |
| Marginal R^2^ / Conditional R^2^ | 0.131 / 0.397 | | | |

Note: Marginal R^2^ indicates variance explained by fixed factors, and conditional R^2^ indicates variance explained by both fixed and random factors.

Table S19. Summaries of LMMs that estimate the effects of condition (subtitles or no-subtitles), school year, and BPVS vocabulary scores on the number of fixations and total time reading in the subtitle and the main scene regions in children. Significant effects are shown in bold font.

|  | **Number of fixations (subtitle region)** | | | | **Total fixation duration**  **(subtitle region)** | | | | **Number of fixations**  **(main scene region)** | | | | **Total fixation duration**  **(main scene region)** | | | |
| --- | --- | --- | --- | --- | --- | --- | --- | --- | --- | --- | --- | --- | --- | --- | --- | --- |
| *Predictors* | *Est* | *CI* | *t* | *p* | *Est* | *CI* | *t* | *p* | *Est* | *CI* | *t* | *p* | *Est* | *CI* | *t* | *p* |
| (Intercept) | 1.43 | 1.18 – 1.68 | 11.27 | **<0.001** | 313.53 | 264.77 – 362.30 | 12.60 | **<0.001** | 4.01 | 3.47 – 4.54 | 14.69 | **<0.001** | 1196.03 | 1033.07 – 1358.98 | 14.39 | **<0.001** |
| condition (subtitles) | 1.23 | 1.22 – 1.24 | 215.34 | **<0.001** | 265.33 | 262.67 – 267.99 | 195.81 | **<0.001** | -0.64 | -0.69 – -0.58 | -21.95 | **<0.001** | -273.03 | -294.00 – -252.06 | -25.52 | **<0.001** |
| school year | 0.03 | -0.03 – 0.10 | 1.08 | 0.279 | 5.68 | -7.73 – 19.08 | 0.83 | 0.406 | 0.00 | -0.07 – 0.08 | 0.03 | 0.976 | -13.35 | -30.50 – 3.80 | -1.53 | 0.127 |
| BPVS | 0.01 | 0.01 – 0.02 | 4.52 | **<0.001** | 2.24 | 1.12 – 3.35 | 3.93 | **<0.001** | -0.00 | -0.01 – 0.00 | -1.45 | 0.147 | -1.33 | -2.76 – 0.11 | -1.81 | 0.070 |
| condition x school year | 0.06 | 0.05 – 0.07 | 11.25 | **<0.001** | 10.64 | 8.23 – 13.04 | 8.66 | **<0.001** | -0.02 | -0.06 – 0.02 | -0.88 | 0.381 | -13.90 | -27.02 – -0.78 | -2.08 | **0.038** |
| condition x BPVS | 0.01 | 0.01 – 0.01 | 23.08 | **<0.001** | 1.93 | 1.73 – 2.14 | 18.75 | **<0.001** | -0.01 | -0.01 – -0.00 | -3.32 | **0.001** | -2.44 | -3.55 – -1.34 | -4.35 | **<0.001** |
| **Random Effects** | | | | | | | | | | | | | | | | |
| σ^2^ | 2.12 | | | | 118945.89 | | | | 2.18 | | | | 124928.06 | | | |
| τ_00_ | 0.56 _item_ | | | | 33763.21 _item_ | | | | 3.01 _item_ | | | | 374350.32 _item_ | | | |
|  | 0.21 _participant_ | | | | 9637.29 _participant_ | | | | 0.31 _participant_ | | | | 15846.14 _participant_ | | | |
|  | 0.01 _school_ | | | | 17.70 _school_ | | | |  | | | | 358.35 _school_ | | | |
|  | 0.04 _videofilename_ | | | | 1845.79 _videofilename_ | | | | 0.26 _videofilename_ | | | | 22613.76 _videofilename_ | | | |
| τ_11_ | 0.01 _item.school_year_ | | | | 416.43 _item.school_year_ | | | | 0.13 _item.condition_ | | | | 21885.60 _item.condition_ | | | |
|  |  | | | |  | | | | 0.00 _item.school_year_ | | | | 698.03 _item.school_yearc_ | | | |
|  |  | | | |  | | | | 0.08 _participant.condition_ | | | | 9462.09 _participant.condition_ | | | |
| ρ_01_ | 0.52 _item_ | | | | 0.33 _item_ | | | | -0.53 _item.condition_ | | | | -0.64 _item.condition_ | | | |
|  |  | | | |  | | | | -0.09 _item.school_year_ | | | | -0.29 _item.school_year_ | | | |
|  |  | | | |  | | | | 0.15 _participant_ | | | | 0.68 _participant_ | | | |
| ICC | 0.29 | | | | 0.28 | | | | 0.64 | | | | 0.78 | | | |
| N | 180 _participant_ | | | | 180 _participant_ | | | | 180 _participant_ | | | | 180 _participant_ | | | |
|  | 365 _item_ | | | | 365 _item_ | | | | 365 _item_ | | | | 365 _item_ | | | |
|  | 4 _videofilename_ | | | | 4 _videofilename_ | | | | 4 _videofilename_ | | | | 4 _videofilename_ | | | |
|  | 5 _school_ | | | | 5 _school_ | | | |  | | | | 5 _school_ | | | |
| Observations | 65700 | | | | 65700 | | | | 65700 | | | | 65700 | | | |
| Marginal R^2^ / Conditional R^2^ | 0.363 / 0.546 | | | | 0.316 / 0.508 | | | | 0.068 / 0.661 | | | | 0.125 / 0.809 | | | |

Note: “Item” refers to “subtitle”. Marginal R^2^ indicates variance explained by fixed factors, and conditional R^2^ indicates variance explained by both fixed and random factors.

In the subtitle region, the analysis of condition-by-year interaction showed a significant effect of school year in the subtitle condition (*β* = 0.09; 95% CI = [0.03, 0.16]; *t* = 2.86; *p* < .001 for the number of fixations; *β* = 16.31; 95% CI = [2.70, 29.93]; *t* = 2.35; *p* = .02 for fixation duration) but not in the no-subtitle condition (*β* = -0.02; 95% CI = [-0.09, 0.04]; *t* = -0.72; *p* = .47 for the number of fixations; *β* = -4.96; 95% CI = [-18.58, 8.66]; *t* = -0.71; *p* = .48 for fixation duration). Similarly, the analysis of condition-by-BPVS interaction showed a significant effect of BPVS in the subtitle condition (*β* = 0.02; 95% CI = [0.02, 0.03]; *t* = 8.14; *p* < .001 for the number of fixations; *β* = 4.17; 95% CI = [3.04, 5.30]; *t* = 7.22; *p* < .001 for fixation duration) but not in the no-subtitle condition (*β* = 0.00; 95% CI = [-0.00, 0.01]; *t* = 0.78; *p* = .43 for the number of fixations; *β* = 0.30; 95% CI = [-0.83, 1.43]; *t* = 0.52; *p* = .60 for fixation duration).

Table S20. Summaries of GLMMs and GLMs that estimate the condition (subtitles or no-subtitles), school year, and BPVS vocabulary scores on the number of crossovers between the subtitle and the main scene regions in children. Significant effects are shown in bold font.

|  | **Probability of a crossover**  **(interaction)** | | | | **Probability of a crossover**  **(nested contrasts)** | | | |
| --- | --- | --- | --- | --- | --- | --- | --- | --- |
| *Predictors* | *L-O* | *CI* | *z* | *p* | *L-O* | *CI* | *z* | *p* |
| (Intercept) | -2.13 | -2.15 – -2.12 | -270.12 | **<0.001** | -2.13 | -2.15 – -2.12 | -270.12 | **<0.001** |
| condition (subtitles) | 0.98 | 0.96 – 1.00 | 123.99 | **<0.001** | 0.98 | 0.96 – 1.00 | 123.99 | **<0.001** |
| school year | 0.02 | 0.01 – 0.03 | 2.70 | **0.007** |  |  |  |  |
| BPVS | 0.01 | 0.00 – 0.01 | 8.71 | **<0.001** |  |  |  |  |
| condition x school year | 0.08 | 0.07 – 0.10 | 11.59 | **<0.001** |  |  |  |  |
| condition x BPVS | 0.00 | -0.00 – 0.00 | 1.76 | 0.079 |  |  |  |  |
| subtitles : school year |  |  |  |  | 0.10 | 0.09 – 0.11 | 18.96 | **<0.001** |
| no-subtitles : school year |  |  |  |  | -0.06 | -0.09 – -0.04 | -4.79 | **<0.001** |
| subtitles : BPVS |  |  |  |  | 0.01 | 0.01 – 0.01 | 13.79 | **<0.001** |
| no-subtitles : BPVS |  |  |  |  | 0.00 | 0.00 – 0.01 | 3.76 | **<0.001** |
| Observations | 271472 | | | | 271472 | | | |
| R^2^ Tjur | 0.085 | | | | 0.085 | | | |

Note: “L-O” refers to “log odds”. “R^2^ Tjur” is the coefficient of discrimination (equals 1 for a model with perfect discriminating power).

Table S21. Summaries of GLMMs that estimate the effects of school year and BPVS vocabulary scores on the probability of skipping subtitles in children. Significant effects are shown in bold font.

|  | **Probability of skipping subtitles** | | | |
| --- | --- | --- | --- | --- |
| *Predictors* | *L-O* | *CI* | *z* | *p* |
| (Intercept) | -1.60 | -2.15 – -1.05 | -5.69 | **<0.001** |
| school year | -0.30 | -0.46 – -0.13 | -3.56 | **<0.001** |
| BPVS | -0.03 | -0.05 – -0.02 | -4.29 | **<0.001** |
| **Random Effects** | | | | |
| σ^2^ | 3.29 | | | |
| τ_00_ _item_ | 1.60 | | | |
| τ_00_ _participant_ | 1.43 | | | |
| τ_00_ _school_ | 0.02 | | | |
| τ_00_ _videofilename_ | 0.23 | | | |
| ICC | 0.50 | | | |
| N _participant_ | 180 | | | |
| N _item_ | 365 | | | |
| N _videofilename_ | 4 | | | |
| N _school_ | 5 | | | |
| Observations | 32850 | | | |
| Marginal R^2^ / Conditional R^2^ | 0.151 / 0.574 | | | |

Note: “Item” refers to “subtitle”. “L-O” refers to “log odds”. Marginal R^2^ indicates variance explained by fixed factors, and conditional R^2^ indicates variance explained by both fixed and random factors.

Table S22. Summaries of LMMs that estimate the effects of word length and frequency and their interaction with school year and BPVS vocabulary scores on fixation durations in children. Significant effects are shown in bold font.

|  | **Gaze duration** | | | | **Total fixation duration** | | | |
| --- | --- | --- | --- | --- | --- | --- | --- | --- |
| *Predictors* | *Est.* | *CI* | *t* | *p* | *Est.* | *CI* | *t* | *p* |
| (Intercept) | 5.54 | 5.50 – 5.57 | 336.47 | **<0.001** | 5.65 | 5.62 – 5.68 | 390.03 | **<0.001** |
| length | 0.03 | 0.03 – 0.04 | 10.22 | **<0.001** | 0.04 | 0.04 – 0.05 | 10.62 | **<0.001** |
| frequency | -0.02 | -0.02 – -0.01 | -8.95 | **<0.001** | -0.02 | -0.02 – -0.01 | -8.83 | **<0.001** |
| school year | -0.04 | -0.06 – -0.02 | -3.80 | **<0.001** | -0.04 | -0.06 – -0.02 | -3.84 | **<0.001** |
| BPVS | -0.00 | -0.00 – -0.00 | -3.37 | **0.001** | -0.00 | -0.00 – -0.00 | -2.50 | **0.012** |
| school year x length | 0.00 | -0.00 – 0.00 | 0.04 | 0.971 | 0.00 | -0.00 – 0.00 | 0.60 | 0.549 |
| school year x frequency | 0.00 | -0.00 – 0.00 | 0.93 | 0.350 | 0.00 | 0.00 – 0.00 | 2.31 | **0.021** |
| BPVS x length | -0.00 | -0.00 – 0.00 | -1.56 | 0.118 | -0.00 | -0.00 – 0.00 | -0.55 | 0.582 |
| BPVS x frequency | 0.00 | -0.00 – 0.00 | 1.76 | 0.079 | 0.00 | -0.00 – 0.00 | 1.37 | 0.170 |
| **Random Effects** | | | | | | | | |
| σ^2^ | 0.24 | | | | 0.29 | | | |
| τ_00_ | 0.02 _item_ | | | | 0.03 _item_ | | | |
|  | 0.02 _participant_ | | | | 0.02 _participant_ | | | |
|  | 0.00 _school_ | | | | 0.00 _school_ | | | |
| τ_11_ | 0.00 _item.school_year_ | | | | 0.00 _participant.length_ | | | |
|  | 0.00 _participant.length_ | | | | 0.00 _participant.logfreq_ | | | |
|  | 0.00 _participant.logfreq_ | | | |  | | | |
| ρ_01_ | -0.02 _item_ | | | | 0.16 _participant.length_ | | | |
|  | 0.20 _participant.length_ | | | | -0.18 _participant.logfreq_ | | | |
|  | -0.22 _participant.logfreq_ | | | |  | | | |
| ICC | 0.15 | | | | 0.16 | | | |
| N | 180 _participant_ | | | | 180 _participant_ | | | |
|  | 873 _item_ | | | | 873 _item_ | | | |
|  | 5 _school_ | | | | 5 _school_ | | | |
| Observations | 53920 | | | | 53920 | | | |
| Marginal R^2^ / Conditional R^2^ | 0.075 / 0.217 | | | | 0.074 / 0.221 | | | |

Note: “Item” refers to “word”. Marginal R^2^ indicates variance explained by fixed factors, and conditional R^2^ indicates variance explained by both fixed and random factors.

Table S23. Summaries of LMMs and a GLM that estimate the effects of school year and BPVS vocabulary scores on the number of fixations, mean fixation duration, and skipping probabilities of words in children. Significant effects are shown in bold font.

|  | **Number of fixations** | | | | **Mean fixation duration** | | | | **Probability of skipping** | | | | **Probability of skipping**  **(word relative position)** | | | |
| --- | --- | --- | --- | --- | --- | --- | --- | --- | --- | --- | --- | --- | --- | --- | --- | --- |
| *Predictors* | *Est.* | *CI* | *t* | *p* | *Est.* | *CI* | *t* | *p* | *L-O* | *CI* | *z* | *p* | *L-O* | *CI* | *z* | *p* |
| (Intercept) | 1.41 | 1.38 – 1.44 | 98.00 | **<0.001** | 5.42 | 5.38 – 5.45 | 279.45 | **<0.001** | 0.16 | 0.15 – 0.17 | 27.59 | **<0.001** | 0.16 | 0.15 – 0.18 | 27.75 | **<0.001** |
| school year | -0.02 | -0.04 – -0.01 | -2.55 | **0.011** | -0.03 | -0.05 – -0.01 | -2.47 | **0.013** | -0.02 | -0.03 – -0.00 | -2.85 | **0.004** | -0.02 | -0.03 – -0.01 | -3.06 | **0.002** |
| BPVS | -0.00 | -0.00 – 0.00 | -0.88 | 0.380 | -0.00 | -0.00 – -0.00 | -2.39 | **0.017** | -0.01 | -0.01 – -0.01 | -15.09 | **<0.001** | -0.01 | -0.01 – -0.01 | -15.55 | **<0.001** |
| word relative position in a sentence |  |  |  |  |  |  |  |  |  |  |  |  | -0.17 | -0.17 – -0.16 | -60.15 | **<0.001** |
| word relative position x BPVS |  |  |  |  |  |  |  |  |  |  |  |  | 0.00 | 0.00 – 0.00 | 2.11 | **0.035** |
| word relative position x school year |  |  |  |  |  |  |  |  |  |  |  |  | 0.01 | 0.00 – 0.01 | 2.44 | **0.015** |
| **Random Effects** | | | | | | | | | | | | | | | | |
| σ^2^ | 0.44 | | | | 0.18 | | | |  | | | |  | | | |
| τ_00_ | 0.08 _item_ | | | | 0.01 _item_ | | | |  | | | |  | | | |
|  | 0.02 _participant_ | | | | 0.02 _participant_ | | | |  | | | |  | | | |
|  |  | | | | 0.00 _school_ | | | |  | | | |  | | | |
| τ_11_ | 0.00 _item.school_year_ | | | | 0.00 _item.school_year_ | | | |  | | | |  | | | |
| ρ_01_ | -0.44 _item_ | | | | -0.03 _item_ | | | |  | | | |  | | | |
| ICC | 0.18 | | | | 0.17 | | | |  | | | |  | | | |
| N | 180 _participant_ | | | | 180 _participant_ | | | |  | | | |  | | | |
|  | 873 _item_ | | | | 873 _item_ | | | |  | | | |  | | | |
|  |  | | | | 5 _school_ | | | |  | | | |  | | | |
| Observations | 53920 | | | | 53920 | | | | 118716 | | | | 118716 | | | |
| Marginal R^2^ / Conditional R^2^ | 0.004 / 0.184 | | | | 0.022 / 0.191 | | | | 0.005 | | | | 0.037 | | | |

Note: “Item” refers to “word”. “L-O” refers to “log odds”. Marginal R^2^ indicates variance explained by fixed factors, and conditional R^2^ indicates variance explained by both fixed and random factors.

Table S24. Summaries of LMMs that estimate the effects of condition (subtitles or no-subtitles), school year, and BPVS vocabulary scores on response accuracy to comprehension questions in children. Significant effects are shown in bold font.

|  | **Accuracy** | | | |
| --- | --- | --- | --- | --- |
| *Predictors* | *Est.* | *CI* | *t* | *p* |
| (Intercept) | 15.38 | 14.48 – 16.28 | 33.44 | **<0.001** |
| condition (subtitles) | 0.08 | -0.09 – 0.25 | 0.96 | 0.339 |
| school year | 0.29 | -0.04 – 0.63 | 1.72 | 0.086 |
| BPVS | 0.08 | 0.06 – 0.11 | 7.52 | **<0.001** |
| condition x school year | 0.03 | -0.12 – 0.18 | 0.39 | 0.699 |
| condition x BPVS | -0.00 | -0.01 – 0.01 | -0.25 | 0.806 |
| **Random Effects** | | | | |
| σ^2^ | 4.34 | | | |
| τ_00_ _participant_ | 2.73 | | | |
| τ_00_ _videofilename_ | 0.76 | | | |
| τ_11_ _participant.condition_ | 0.24 | | | |
| τ_11_ _videofilename.school_year_ | 0.05 | | | |
| ρ_01_ _participant_ | 0.10 | | | |
| ρ_01_ _videofilename_ | -1.00 | | | |
| ICC | 0.47 | | | |
| N _participant_ | 180 | | | |
| N _videofilename_ | 4 | | | |
| Observations | 720 | | | |
| Marginal R^2^ / Conditional R^2^ | 0.351 / 0.657 | | | |

Note: Marginal R^2^ indicates variance explained by fixed factors, and conditional R^2^ indicates variance explained by both fixed and random factors.
